# Catechin and curcumin interact with S protein of SARS-CoV2 and ACE2 of human cell membrane: insights from computational studies

**DOI:** 10.1038/s41598-021-81462-7

**Published:** 2021-01-21

**Authors:** Atala B. Jena, Namrata Kanungo, Vinayak Nayak, G. B. N. Chainy, Jagneshwar Dandapat

**Affiliations:** 1 Centre of Excellence in Integrated Omics and Computational Biology, Utkal University, Bhubaneswar, 751004 Odisha India; 2 Post Graduate Department of Biotechnology, Utkal University, Bhubaneswar, 751004 Odisha India

**Keywords:** Computational biology and bioinformatics, Structural biology

## Abstract

The recent outbreak of the coronavirus (SARS-CoV2) is an unprecedented threat to human health and society across the globe. In this context, development of suitable interventions is the need of the hour. The viral spike protein (S Protein) and the cognate host cell receptor ACE2 can be considered as effective and appropriate targets for interventions. It is evident from the present computational study, that catechin and curcumin, not only exhibit strong binding affinity to viral S Protein and host receptor ACE2 but also to their complex (receptor-binding domain (RBD) of the spike protein of SARS-CoV2 and ACE2; RBD/ACE2-complex). The binding affinity values of catechin and curcumin for the S protein, ACE2 and RBD/ACE2-complex are − 10.5 and − 7.9 kcal/mol; − 8.9 and − 7.8 kcal/mol; and − 9.1 and − 7.6 kcal/mol, respectively. Curcumin directly binds to the receptor binding domain (RBD) of viral S Protein. Molecular simulation study over a period of 100 ns further substantiates that such interaction within RBD site of S Protein occurs during 40–100 ns out of 100 ns simulation trajectory. Contrary to this, catechin binds with amino acid residues present near the RBD site of S Protein and causes fluctuation in the amino acid residues of the RBD and its near proximity. Both catechin and curcumin bind the interface of ‘RBD/ACE2-complex’ and intervene in causing fluctuation of the alpha helices and beta-strands of the protein complex. Protein–protein interaction studies in presence of curcumin or catechin also corroborate the above findings suggesting the efficacy of these two polyphenols in hindering the formation of S Protein-ACE2 complex. In conclusion, this computational study for the first time predicts the possibility of above two polyphenols for therapeutic strategy against SARS-CoV2.

## Introduction

**Figure 1:**
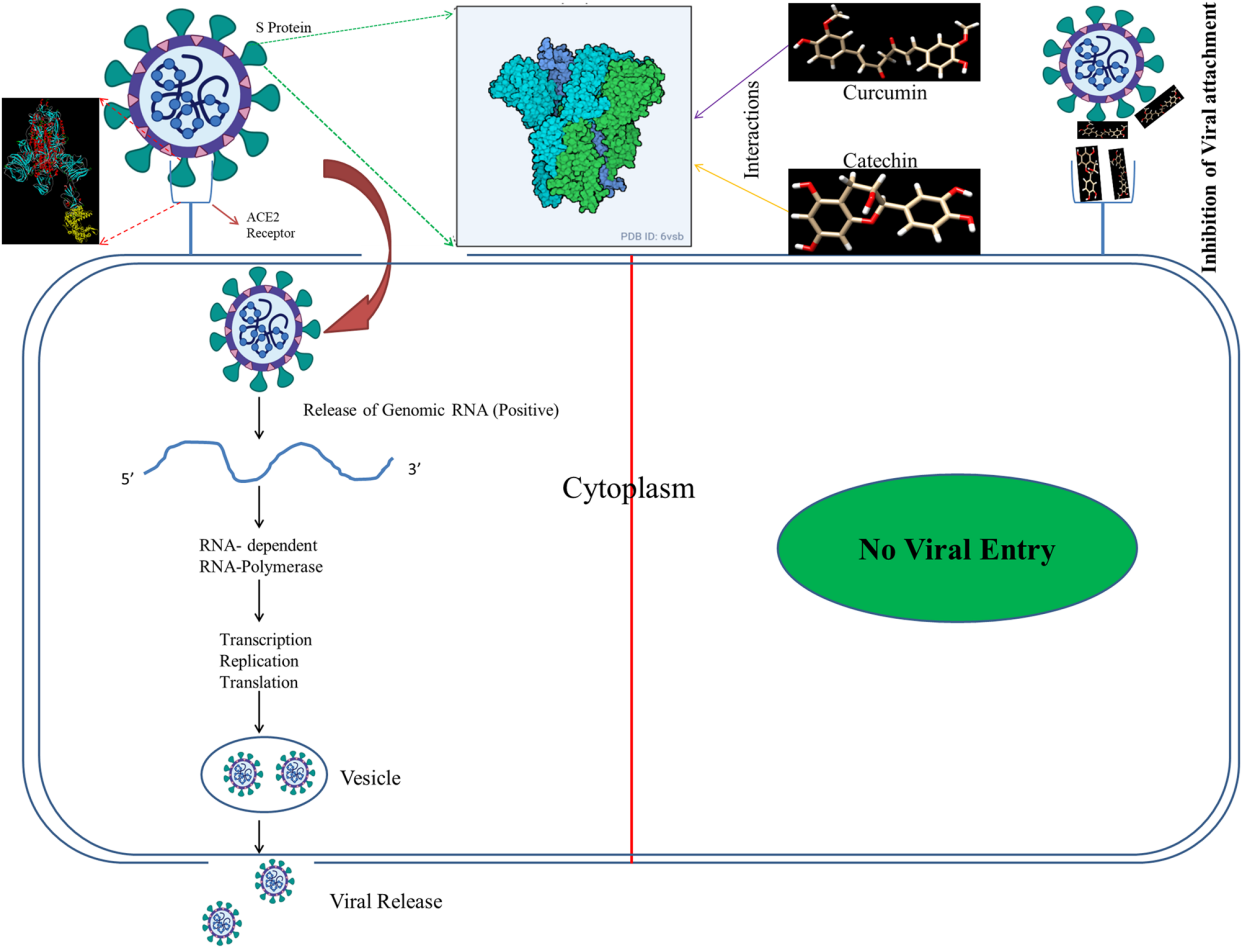
Pictorial depiction of the binding of viral S Protein with the ACE2 cellular receptor. The presence of catechin and curcumin inhibits S Protein and ACE2 interaction and eventually prevents viral entry.

SARC-CoV2, an enveloped single stranded positive sense RNA virus belonging to family *Coronaviridae* is a matter of global concern^1,2^. WHO declared its outbreak as pandemic due to its high rate of transmission, rapid spread over several countries and unavailability of specific vaccines or medication to treat it^3^. Phylogenetically SARS CoV2 belongs to order *Nidovirales*^4^ and grouped under Beta coronavirus, with a genome size of ~ 30 kilobases, which codes for different structural and accessory proteins^4,5^. The general morphology of coronavirus includes different structural proteins such as spike (S) protein, envelope (E) protein, membrane (M) protein and the nucleocapsid (N) protein^6^. Corona virus invades human cells through binding of its distinct surface spike protein (glycoprotein in nature) with a receptor protein (Angiotensin Converting Enzyme 2 (ACE2)) present on the membrane of human cells. This mediates receptor attachment and viral-host cell membrane fusion (Fig. 1). The S protein is a transmembrane protein with N- exo and C- endo terminals. The N terminal S_1_ subunit contains Receptor Binding Domain (RBD) while the C terminal S_2_ subunit induces membrane fusion^7^ (Supplementary Fig. S1). Fusion of virus with human cells is resulted due to the binding of S_1_ subunit of viral protein S to human cell receptors^7,8^. On the other hand, consequent upon endocytosis of the virus, the S_2_ subunit which is characterised by Heptad Repeats (HR) regions that assembles into an intra-hairpin helical structure with six helix bundle promotes the membrane fusion process inside the host cell^9,10^. Recently, it has been reported that receptor binding domain of S Protein of SARS-CoV2 is more or less similar to that of SARS-CoV, despite amino acid variation at some key residues^2^. This suggests that the virus can also target ACE2, a monomeric membrane bound protein of human cells^11,12^. Therefore, it is presumed that ACE2, the cognate receptor of corona virus present on the cell membrane of host cells can also be a specific target to prevent the viral entry^13^.

Several recent studies have suggested that natural polyphenolic compounds like catechins (GTCs; Green Tea Catechins) and curcumin (diferuloylmethane; from turmeric) have antiviral activities against a broad spectrum of viruses such as Human Immunodeficiency Virus (HIV), Herpes Simplex Virus, Influenza Virus, Hepatitis B and C Viruses (HBV and HCV respectively)^14^, Adenovirus^15^ and Chikungunya virus (CHIKV)^16^. Diverse mechanisms have been suggested to explain the antiviral activities of both the polyphenolic compounds. For example, GTCs have been documented to be a potential suppressor of viral entry and its replication^17–21^, while curcumin has been demonstrated as a potent inhibitor of monophosphate dehydrogenase, a rate limiting enzyme in the de novo synthesis of guanine nucleotide^22^. Further, it has also been observed that GTCs and curcumin inhibit the expression of ACE2, as evident from animal studies^23,24^.

Although catechin and curcumin have been reported to bind with various proteins of viral and human origin, there is not much information on interaction of polyphenols with S protein of the coronavirus and its cognate receptor, ACE2 of host cells till date. With this back drop, the present study has been designed to examine interaction of catechin and curcumin with S protein of the virus and its cognate receptor ACE2 of host cells employing computational methods. Computational approaches (Molecular docking and simulation) are the first and foremost choice of scientists to prophesize apparent binding modes and affinities of ligands for macromolecules before experimental studies which are indeed expensive and time consuming^25^. In addition, improvement in speed, reliability and accuracy of computational docking methods in last few years have made it a suitable choice to design structure-based drugs^26,27^. The present study incorporates results of molecular docking of catechin and curcumin with the S Protein of corona virus as well as with ACE2 of host cells, a cognate receptor for viral S Protein. In addition, binding affinities and molecular simulation studies of catechin and curcumin with the ‘RBD/ACE2-complex’ indicate that both the polyphenols cause considerable alteration in the structure of the complex.

## Materials and methods

### Sequence analysis

The Cryo-EM structure of SARS-CoV2 S Protein (PDB ID-6vsb) and X-Ray diffraction structure of ACE2 (PDB ID-1r42) with resolution of 3.46 Å and 2.2 Å, respectively, were retrieved from PDB database. The Cryo-EM structure of ‘RBD/ACE2-complex’ (PDB ID-6LZG) (RBD of S Protein with ACE2) with resolution 2.50 Å was obtained from PDB database. The complex structure is basically composed of two polypeptides, chain-A (Angiotensin-converting enzyme having 596 amino acid residues) and chain-B (Spike glycoprotein having 209 amino acid residues). The FASTA sequence of S Protein of 2019-nCoV, HCoV-229E, MERS-CoV, HCoV-NL63, SARS-CoV were also retrieved for multiple sequence alignment analysis. The alignment results of SARS-CoV2 portrayed that all the three chains of S Protein have identical amino acid sequences. Therefore, only one chain was taken for secondary structure analysis and prediction of physicochemical properties.

### Protein–protein docking

An automated rigid body docking tool, **clusPro2.0**^28^ was used for S Protein–ACE2 protein docking analysis in presence and absence of curcumin and catechin. This tool helps in screening docked conformations with respect to their clustering properties, taking into consideration different protein parameters. The selection of the filtered conformations was based on the assessment of empirical free energy. Both the lowest desolvation and electrostatic energies were taken into account for the evaluation of free energy. ClusPro is accessible at https://cluspro.bu.edu/publications.php. Piper being a FFT-based rigid docking tool, serves the ClusPro clustering program for detecting native sites by providing 1000 low energy outcomes.

### Molecular docking analysis of S Protein, ACE2 and the ‘RBD/ACE2-complex’ with catechin and curcumin

Evaluation of binding free energy of S Protein, ACE2 and ‘RBD/ACE2-complex’ with catechin and curcumin was done through molecular docking program AutoDock Tools 1.5.6. The canonical SMILES id of catechin (Catechin–Gallocatechin–Catechin) and curcumin were obtained from PubChem database (https://pubchem.ncbi.nlm.nih.gov/). Conversion to 3D structures was done using CHIMERA 1.11.2^29^. Binding affinity of S Protein, ACE2 and ‘RBD/ACE2-complex’ with catechin and curcumin were examined using Auto Dock Vina 1.1.2^30^. Various parameters such as binding affinity, receptor interacting atom, receptor pocket atom, receptor ligand interaction site, atomic contact energy (ACE) and side amino acid residues were studied to recognise the binding site of S Protein, ACE2 and ‘RBD/ACE2-complex’. The results of docking studies were visualised and analysed by Discovery Studio 2017 R2 Client^31^.

### Molecular simulation analysis

The chemically unstandardized 2D structures of ligands, catechin and curcumin were taken up from PubChem database (https://pubchem.ncbi.nlm.nih.gov/). Ligand files can be switched to properly standardised and extrapolated 3D structures by LigPrep^32^. LigPrep plays a major role in conversion of 3D structures to consequently lower energy structures which can be used by Glide^33^. This minimisation of structures is done using OPLS3e force field. Each input structure generates multiple output structures due to different stereochemistry, protonation states, tautomer’s and ring conformations. In the ligand output file specifications are made for production of one low energy ring conformation per ligand. Grid-based Ligand Docking with Energetics (GLIDE) module in Schrodinger software was used for the formation of S Protein–curcumin and S Protein–catechin complex. Also catechin and curcumin were individually complexed with the ‘RBD/ACE2-complex’. Desmond software was used for carrying out molecular dynamics simulations, Root Mean Square Deviations (RMSD) and atomic fluctuation through Root Mean Square Fluctuation (RMSF) studies. For conducting explicit solvent simulations with periodic marginal conditions, different tools such as cubic, orthorhombic, truncated octahedron, rhombic dodecahedron and other arbitrary simulation boxes are used. Prior to 100 ns production run, 8-staged stabilization run was conducted which includes primarily task, then simulations in Brownian Dynamics with NVT at T = 10 K, small time steps, restraints on solute heavy atoms for 100 ps and followed by repetition of the above stage but with restraints on solute heavy atoms for 12 ps. The stage 4 was carried out in a similar manner to the previous one at NPT instead of NVT followed by focus on solvate pocket. The stage 6 is the same as that of stage 4. The next stage involved simulation at NPT for 24 ps with no restraints. Finally, simulations were done.

Here in this study, MD simulations were conducted notably for the top two identified hits to analyse the stability of the ligand receptor complex for 100 ns. Stability of docked complexes 2019-nCoV spike glycoprotein–curcumin and 2019-nCoV spike glycoprotein–catechin are simulated till 100 ns simulation time by performing Molecular Dynamics (MD) simulations using system builder of Desmond^34^ implemented in Maestro^35^ with OPLS3e force field. Simulations were also conducted for both the ligands with ‘RBD/ACE2-complex’. The system for ‘S Protein–curcumin’ and ‘S Protein–catechin’ were immersed in a water filled cubic box of 10 Å spacing containing 63,985 (approximately) water molecules with system builder of the Desmond in the Maestro program. Similarly, for ‘RBD/ACE2-complex’ with catechin and curcumin approximately 26,850 water molecules were taken using extended simple point charge (SPC). Neutralisation of the docked complex was done by the addition of 4 Na^+^ ions (1.137 mM concentration) into the system for S Protein and curcumin. 6 Na^+^ ions (1.706 mM concentration) were added for neutralisation of S Protein and catechin. Similarly, 23 Na^+^ ions (15.575 mM concentration) and 21 Na^+^ ions (14.206 mM concentration) were added for neutralisation of ‘RBD/ACE2-complex’ for catechin and curcumin, respectively.

Molecular Mechanics Generalized Born Surface Area (MM-GBSA) method has been adopted for the calculation of binding free energies of catechin and curcumin with S Protein and ‘RBD/ACE2-complex’, respectively. The more negative value indicates stronger binding as the MM-GBSA is an index of free energy of binding. Prime module^36^ was used to calculate MM-GBSA binding free energy under equilibrated trajectory of molecular dynamics. The decrease in the potential energy during the 100 ns in case of both catechin–S Protein, curcumin–S Protein complexes revealed that the system is stable. Analysis of different conformations acquired over the simulation period of 100 ns is done. For the computation of average change in the displacement of selected atoms in a particular frame with respect to reference frame, Root mean square deviation (RMSD) is estimated for the protein and ligand for 100 ns simulation trajectory.

In order to understand the unbinding trends of both proteins, chain A (Angiotensin-converting enzyme 2) and chain B (RBD of Spike glycoprotein) and to analyse the consistency of such trends in presence of catechin or curcumin, we carried out non-bonding interaction qualitative analysis by slicing every 5 ns frames from the Molecular dynamics trajectory of 100 ns.

## Results and discussion

### Structural analysis

Prediction of secondary structure S Protein of SARS-CoV2 has been done using SOPMA (Self Optimised Prediction Method with Alignment). The S Protein contains 1288 aa residues comprising 350 α helices (27.17%), 312 β-turns (9.08%) and 509 random coils (39.52%). Through ExPASy ProtParam, the total number of negatively charged (Asp + Glu) and positively charged residues (Arg + Lys) were determined to be 112 and 100, respectively. The aliphatic index was found to be 81.58. The GRAVY (Grand Average of Hydropathicity) scored to − 0.163. The instability index was computed to be 31.58. These features classify S Protein of SARS-CoV2 as a stable structure . It was also revealed through computational studies that the half-life of S Protein is maximum in case of mammals (mammalian reticulocytes-30 h) than yeast (> 20 h) and bacteria (*E. Coli* −  > 10 h).

### Structure alignment

SARS-CoV2 and SARS-CoV were evaluated by TM-align (https://zhanglab.ccmb.med.umich.edu/TM-align/) for comparative structural studies. These two viruses were considered for Structure-Structure superimposition due to maximum sequence similarity. It was observed through structural alignment studies that SARS-CoV2 and SARS-CoV only differ in RBD fragment and remaining part of the structure is identical (Supplementary Fig. S2). It was apparent that SARS-CoV is an ancestor of the newly upsurging virus SARS-CoV2. Nevertheless, some changes were noticed in the RBD fragment of SARS-CoV2 compared to SARS-CoV. The results corroborate an earlier study^2^.

### Protein–protein docking

**Figure 2:**
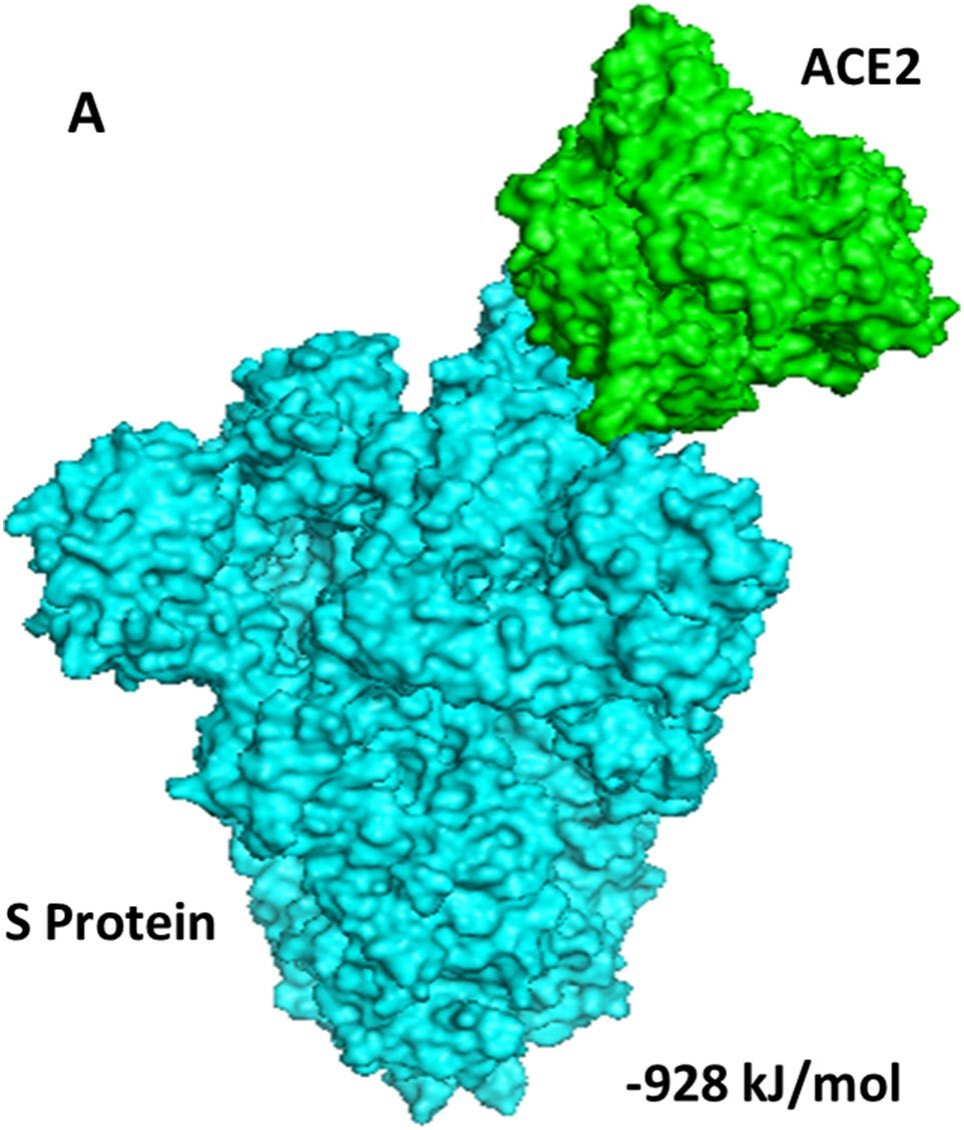
Docked model depicting interaction of S Protein with ACE2 receptor in the absence of polyphenols.

**Figure 3:**
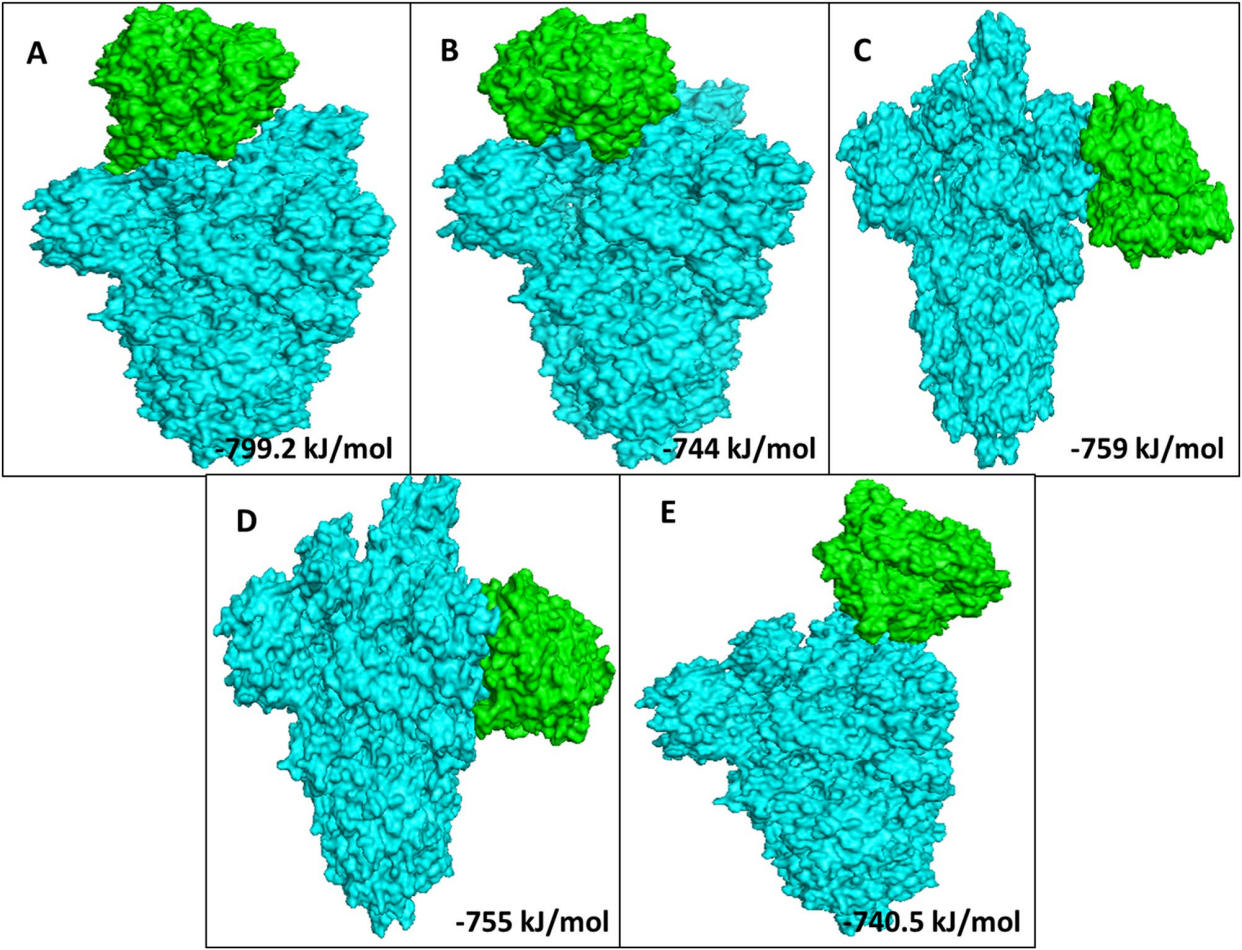
The top 5 docked models displaying interaction of S Protein with ACE2 receptor in the presence of curcumin.

**Figure 4:**
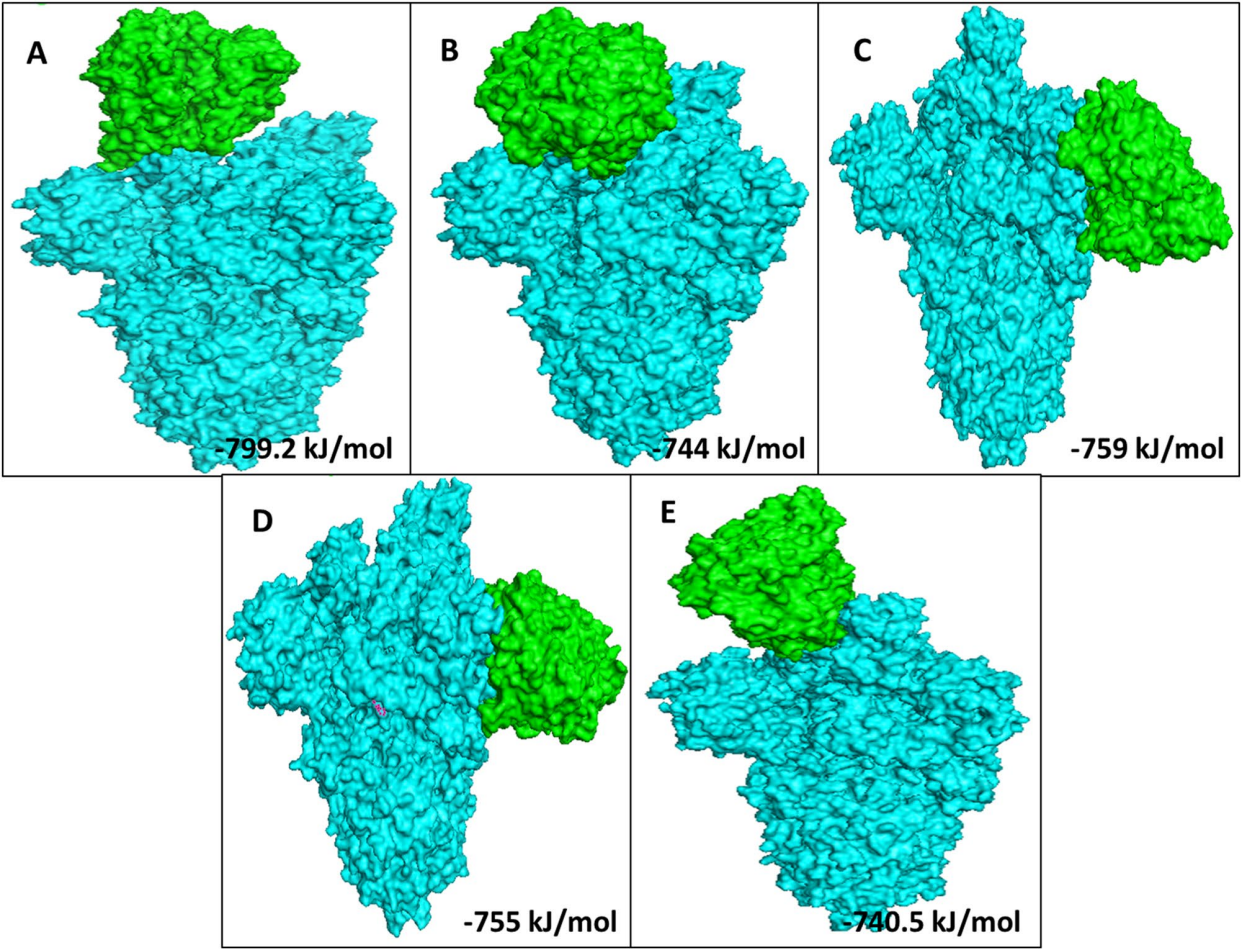
The top 5 docked models displaying interaction of S Protein with ACE2 receptor in the presence of catechin.

Based on the total RMSD value the best 10 docking models with different free energies were obtained from the ClusPro web-server. Out of which, we analysed 5 ClusPro docking models which were selected based on probability of S Protein, S Protein with curcumin and S Protein with catechin to interact with the predicted binding sites of ACE2 with lowest binding energy during such interactions. Average binding energy of all 5 binding positions for S Protein–ACE2 interaction in the absence of curcumin or catechin is − 901.2 kJ/mol (Supplementary Fig. S16). Nevertheless, average binding energy for S Protein–ACE2 in presence of either of the above two polyphenols is − 759.54 kJ/mol (Table 1, Figs. 2, 3, 4).

**Table 1:** Protein–protein interaction depicting 5 lowest binding energy for S Protein–ACE2 complex in the presence or absence of curcumin and catechin.

Macromolecule	Binding positions	Lowest energy (kJ/mol)	Average lowest energy (kJ/mol)
S Protein–ACE2	1	− 928.9	− 901.2
S Protein–ACE2	2	− 923	
S Protein–ACE2	3	− 902.4	
S Protein–ACE2	4	− 853.3	
S Protein–ACE2	5	− 898.4	
S Protein with curcumin–ACE2	1	− 799.2	− 759.54
S Protein with curcumin–ACE2	2	− 744	
S Protein with curcumin–ACE2	3	− 759	
S Protein with curcumin–ACE2	4	− 755	
S Protein with curcumin–ACE2	5	− 740.5	
S Protein with catechin–ACE2	1	− 799.2	− 759.54
S Protein with catechin–ACE2	2	− 744	
S Protein with catechin–ACE2	3	− 759	
S Protein with catechin–ACE2	4	− 755	
S Protein with catechin–ACE2	5	− 740.5

It was observed that during protein–protein interaction binding energy of S Protein–ACE2 decreases in presence of the phytocompounds, (i.e. curcumin or catechin). A significant decline in 141.66 kJ/mol of binding energy was observed during the interaction of S Protein–ACE2 in presence of curcumin or catechin compared to their direct binding. Therefore, it can be presumed that both the compounds are capable of hindering the attachment of RBD site of S Protein to the ACE2 receptor protein. This would indeed pave a way for the utilisation of curcumin or catechin in repurposing/design of effective therapy to prevent the viral entry.

### Molecular docking and simulation analyses

**Figure 5:**
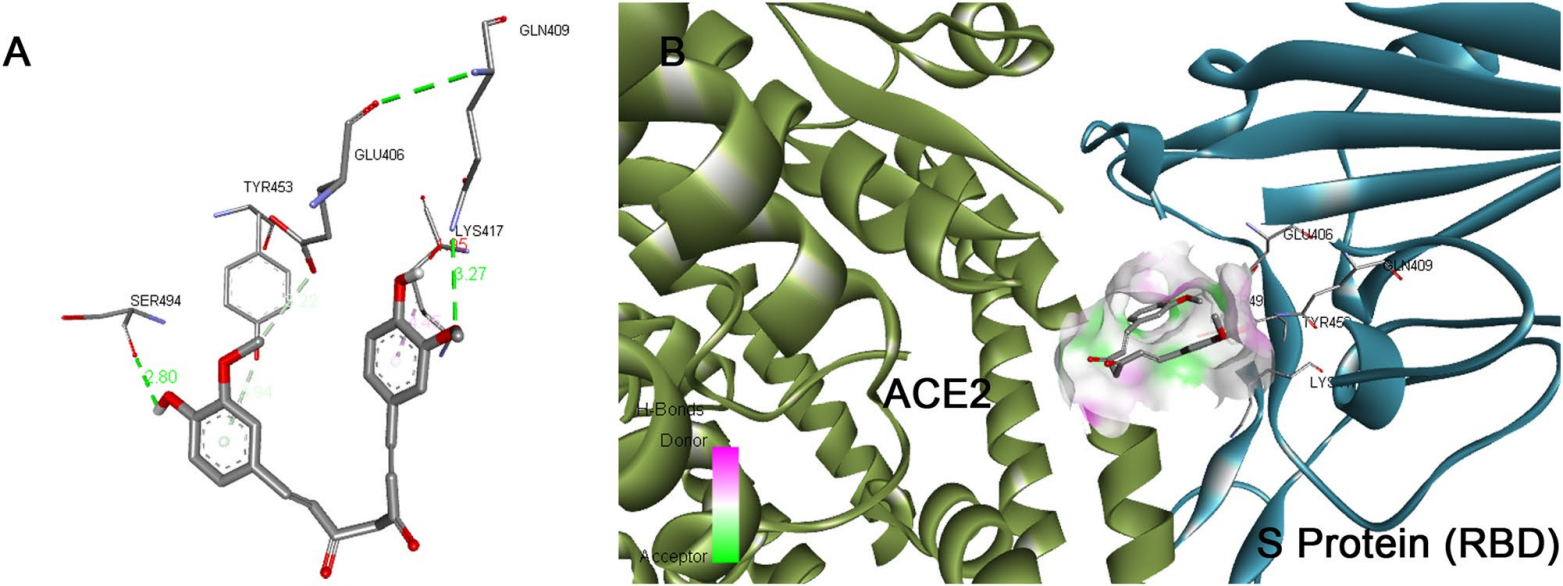
Docked pose of curcumin in the interface of RBD/ACE2-complex. (**A**) Participating amino acids in the interaction of curcumin and RBD/ACE2-complex, (**B**) Several bonds involved in the interaction between curcumin and RBD/ACE2-complex. This figure was produced using Discovery Studio Visualizer (http://accelrys.com/products/collaborative-science/biovia-discovery-studio/visualization-download.php).

**Figure 6:**
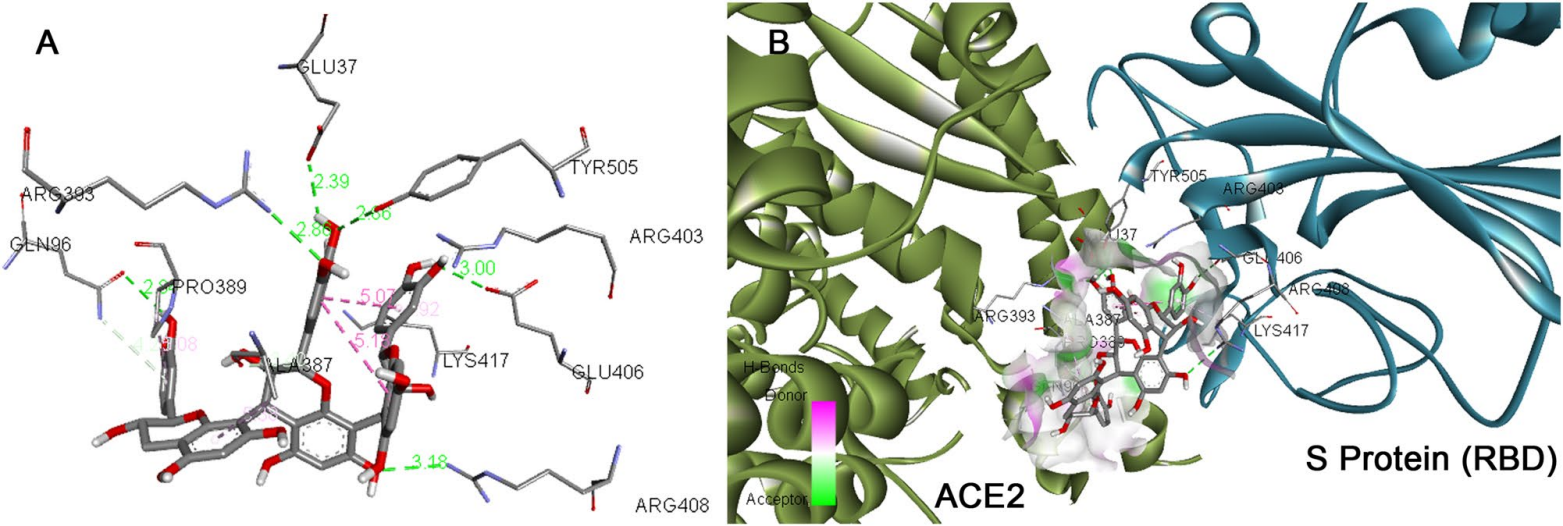
Docked pose of catechin in the interface of RBD/ACE2-complex. (**A**) Participating amino acids in the interaction of catechin and RBD/ACE2-complex, (**B**) Several bonds involved in the interaction between catechin and RBD/ACE2-complex. This figure was produced using Discovery Studio Visualizer (http://accelrys.com/products/collaborative-science/biovia-discovery-studio/visualization-download.php).

The binding modes of catechin and curcumin with S Protein and ACE2 were investigated through Auto DockVina 1.1.2. The binding energy of S Protein with catechin and curcumin scored to be − 10.5 kcal/mol and − 7.9 kcal/mol, respectively (Table 2). The binding affinity of curcumin with ACE2 was noted to be − 7.8 kcal/mol where as that of catechin was found to be − 8.9 kcal/mol (Table 3). Similarly, catechin and curcumin have − 9.1 kcal/mol and − 7.6 kcal/mol binding affinities, respectively, towards the ‘RBD/ACE2-complex’ (Table 4). From the docking scores, it can be deduced that both catechin and curcumin have strong binding affinity for S Protein, ACE2 as well as ‘RBD/ACE2-complex’. The amino acid residues of S Protein, ACE2 as well as ‘RBD/ACE2-complex’ that participate in the formation of different chemical bonds (such as van der Waals force, conventional hydrogen bonds and carbon–hydrogen bonds) with catechin or curcumin differ among proteins and their complex (Figs. 5, 6, Supplementary Figs. S3, S4, S8 and S9). On the other hand, molecular docking investigations have suggested that binding affinities of catechin for S Protein, ACE2 and also ‘RBD/ACE2-complex’ are higher than that of curcumin.

**Table 2:** The binding energy, types of interaction and amino acids involved in the interaction of S protein of SARS-CoV2 with curcumin and catechin.

Protein–Ligand	Binding affinity (kcal/mol)	Types of interaction	Interacting AA Name; AA No;
S Protein–curcumin	− 7.9	Van der waals	Leu546, Gly548, Phe541, Asn856, Leu997, Ser967, Asp571, Ala570, Val976, Thr572
		Conventional hydrogen bond	Asp979, Thr547, Arg1000, Ser975
		Carbon–hydrogen bond	Thr573, Asn978, Cys743
		Pi–donor hydrogen bond	Thr573, Asn978, Cys743
		Pi–Sigma	Leu966
S Protein–catechin	− 10.5	Van der waals	Gln314, Glu309, Lys310, Gly311, Ile664, Lys733, Leu861, Asp950, Gly769, Ala766, Lys304, Thr761
		Conventional hydrogen bond	Tyr313, Thr768, Asn764, Thr302, Gln954, Asp775
		Carbon–hydrogen bond	Ile312
		Pi–sigma	Thr768, Val772, Leu303
		Pi–cation	Arg765
		Pi–alkyl	Ile312, Pro665, Arg765

**Table 3:** The binding energy, types of interaction and amino acids involved in the interaction of human ACE2 receptor with curcumin and catechin.

Receptor–ligand	Binding affinity (kcal/mol)	Types of interaction	Interacting AA Name; AA No;
ACE2–curcumin	− 7.8	Van der waals	Leu591, Lys94, Asn210, Glu564, Glu280, Tyr207, Asp206, Gly205, Tyr196, Ala99, Lys562, Ala396
		Conventional hydrogen bond	Gln102, Trp566
		Carbon–hydrogen bond	Gln98
		Pi–alkyl	Val209, Pro565, Val212
		Pi–sigma	Leu95
ACE2-catechin	− 8.9	Van der waals	Gly66, Asn63, Asn51, Tyr50, Tyr510, Arg514, Ala348, His378, Gly352, Asp350, Ser44, Trp349
		Conventional hydrogen bond	Ser43, Asp382
		Carbon–hydrogen bond	His401, Trp69, Ser47
		Pi–alkyl	Met62, Arg393
		Pi–Pi stacked	His401, Phe390, Phe40
		Pi–Pi T-shaped	His401, Phe390, Phe40
		Unfavourable donor–donor	Arg393

**Table 4:** The binding energy, types of interaction and amino acids involved in the interaction of RBD/ACE2-complex with curcumin and catechin.

Receptor–ligand	Binding affinity (kcal/mol)	Types of interaction	Interacting AA name AA No; For ACE2	Interacting AA name AA No; For S protein
RBD/ACE2-complex with curcumin	− 7.6	Van der waals	Phe390, Asn33, Glu37, His34, Asp38, Lys353, Arg396, Pro389	Arg408, Gly416, Ile418, Tyr505, Gln493, Gly496, Tyr495, Arg403,
		Conventional hydrogen bond		Gln409, Ser494
		Carbon–hydrogen bond		Glu406
		Pi–alkyl		Lys417
		Unfavourable donor–donor		Gln409
		Pi donor hydrogen bond		Tyr453
RBD/ACE2-complex with catechin	− 9.1	Van der waals	Arg559, Gln338, Ser536,Thr92, Leu 29, Val 93, Lys26, Asn33, Ala386, His 34	Gly416, Gln409, Asp405, Leu455, Tyr453
		Conventional hydrogen bond	Arg393, Glu37, Gln96	Glu406, Arg408, Tyr505
		Carbon–hydrogen bond	Ala387	
		Pi–alkyl	Pro389	Lys417
		Pi donor hydrogen bond	Gln96	
		Unfavourable acceptor–acceptor		Glu406
		Unfavourable donor–donor		Arg403

The results from Molecular Simulation data throw a light on the interaction of curcumin with S Protein. The Root Mean Square Deviation (RMSD) of S Protein–curcumin complex was marked to increase for the initial 20 ns and then remained stable up to 100 ns during the simulation trajectory (Supplementary Fig. S5). Local changes along the protein chain were characterised through Root Mean Square Fluctuation (RMSF). The plot indicates curcumin possesses the ability to cause fluctuation of all amino acids of S protein (Supplementary Fig. S6). Table S1 represents S Protein and ligand interactions which clearly depict the resident time of specific amino acid residues participating during the process, including the amino acids in the RBD site which display considerable interaction with curcumin. Keto group of curcumin exhibits a high affinity to Leu335 of RBD site of S Protein forming hydrophobic bonds. Interaction with Leu335 of RBD site of S Protein occurs for 40% of the simulation time (Supplementary Fig. S7). Molecular simulation studies are in good agreement with docking studies. Results suggest that both polyphenols bind to S Protein with high binding energy, however, their binding sites on S Protein differ considerably. Curcumin binds directly to the RBD of S Protein whereas catechin binds to proximity of RBD of S Protein. In addition, catechin causes greater fluctuation in amino acid residues near the RBD site.

The RBD fragment of SARS-CoV2 spans from 319–591 S-residues^37^. From our studies it is deduced that curcumin directly binds to amino acids in this region Leu546, Gly548, Phe541, Asp571, Ala570, Thr572, Thr547, Thr573 whereas, catechin binds to the S Protein in the near proximity of RBD fragment to Gln314, Glu309, Lys310, Gly311, Lys304, Tyr313, Thr302, Ile312, Leu303 and Ile312 residues (Table 2, Supplementary Figs. S3 and S8).

The average change in displacement of atoms in all frames was recorded through Root Mean Square Deviation (RMSD) at 10 ns interval. Although the maximum MM-GBSA binding energy for S Protein–curcumin complex was observed − 58 kcal/mol at 0 and 20 ns, the minimum value was recorded − 47 kcal/mol at 10 and 70 ns. On the other hand, the highest and the lowest MM-GBSA binding energy for S Protein–catechin complex were noticed to be − 59 kcal/mol (at 30 ns) and − 20 kcal/mol (at 60 and 80 ns), respectively (Supplementary Fig. S14). Total MM-GBSA free energy calculation for both polyphenols with S Protein indicates a favourable binding energy for the curcumin (− 53.63 kcal/mol) as compared to catechin (− 34.22 kcal/mol). Average RMSD of both complexes (S Protein–catechin complex and S Protein–curcumin complex) was recorded less than 3 Å after stabilization of S Protein RMSD. On the contrary, RMSD of S Protein–catechin complex was initially unstable for the first 50 ns and stabilised thereafter for the rest of the simulation period (Supplementary Fig. S11). Maximum structural fluctuation was observed in between 300 and 500 amino acid residues and after 1000 amino acids residues of S Protein (Supplementary Fig. S10). The above data supports that S Protein and catechin interaction occurs with amino acid residues of S Protein near the RBD site (319 aa–591 aa)^37^. Amino acid residues Arg634 and Val635 near the RBD site of S Protein have stronger affinity towards hydroxyl group of catechin with 54% and 35%, respectively, out of 100 ns simulation trajectory (Supplementary Fig. S12).

The binding affinity of curcumin with ACE2 was noted to be − 7.8 kcal/mol where as that of catechin was found to be − 8.9 kcal/mol. The binding of curcumin or catechin with ACE2 includes conventional hydrogen Bond, carbon–hydrogen bond and Pi–Sigma interactions. The amino acid residues of the protein that take part in the above interactions vary for both ligands (Supplementary Figs. S4, S9 and Table 3).

**Figure 7:**
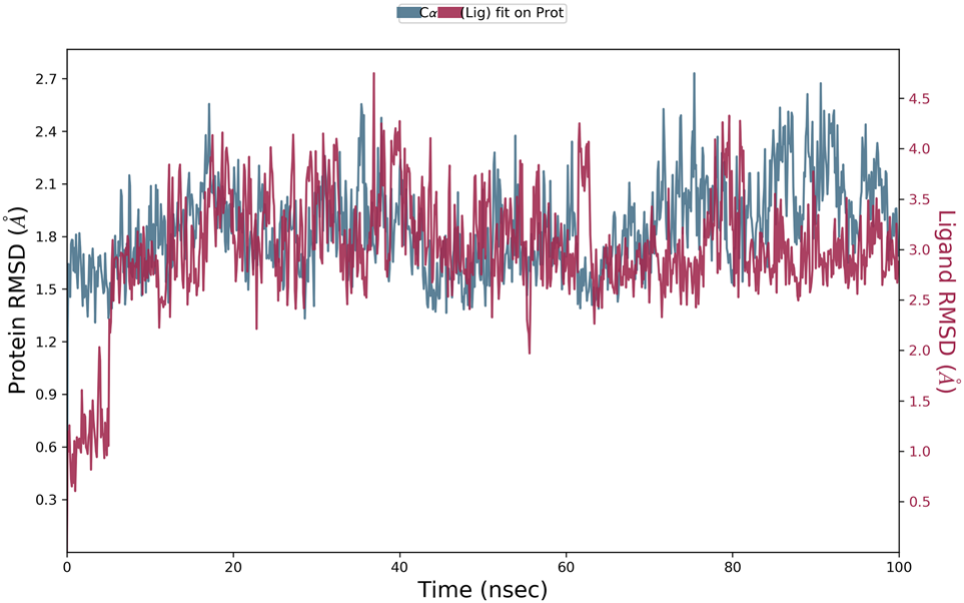
Root mean square deviation (RMSD) plot for interaction of catechin and RBD/ACE2-complex during 0–100 ns of molecular dynamic simulation.

**Figure 8:**
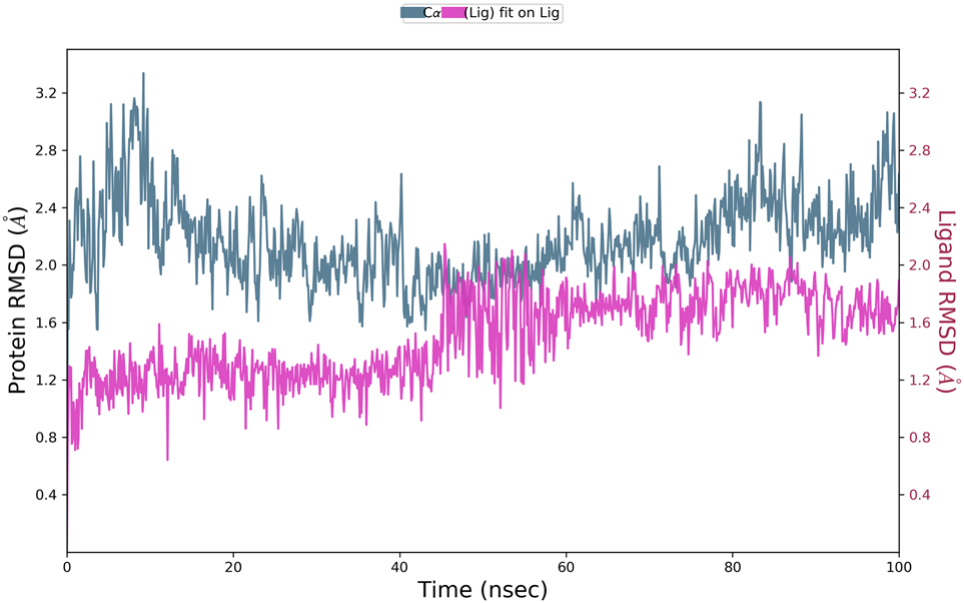
Root mean square deviation (RMSD) plot for interaction of curcumin and RBD/ACE2-complex during 0–100 ns of molecular dynamic simulation.

**Figure 9:**
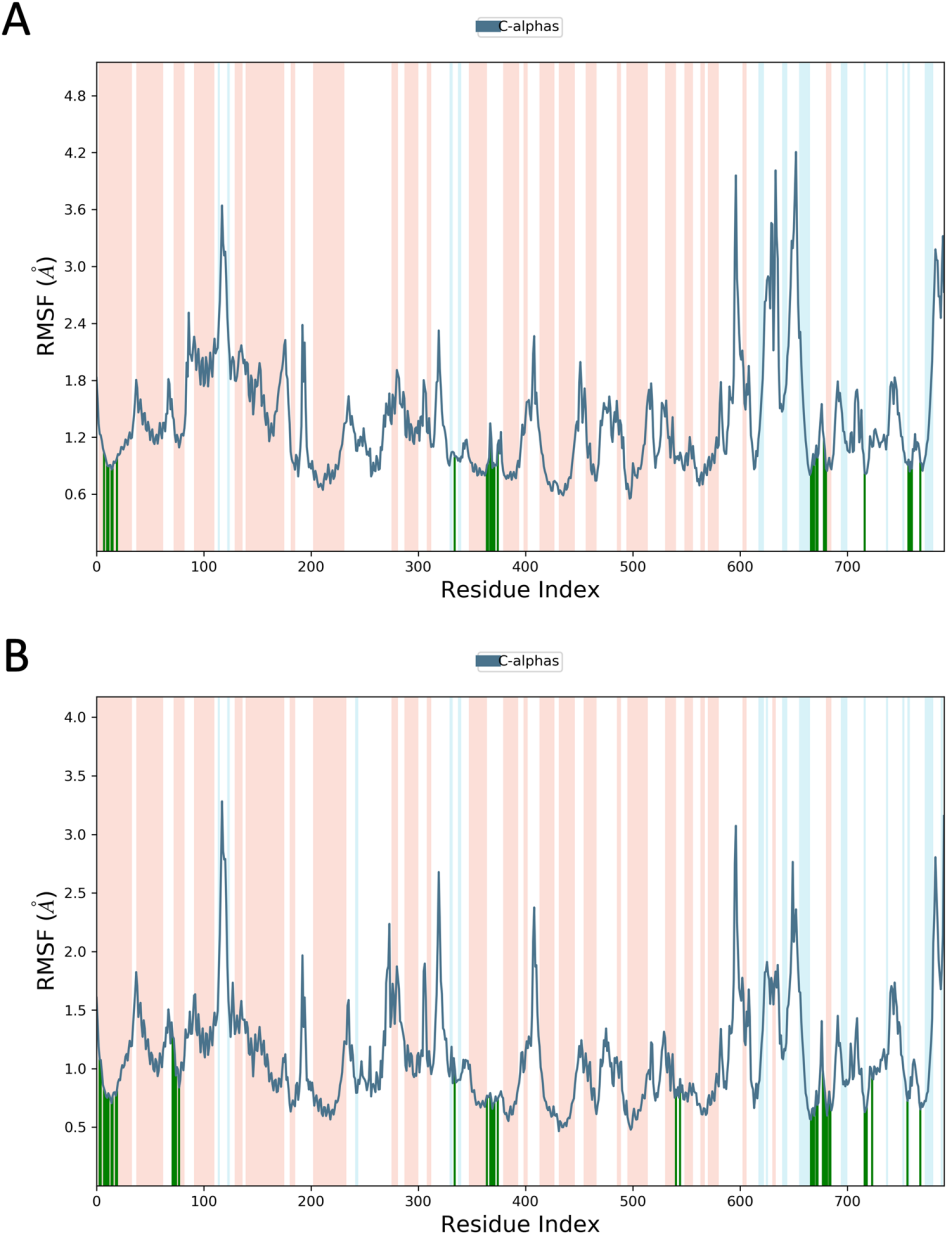
(**A**) RMSF plot depicting curcumin-induced fluctuations in the entire amino acid sequence of RBD/ACE2-complex during 100 ns MD simulations. (**B**) RMSF plot depicting catechin induced fluctuations in the entire amino acid sequence of RBD/ACE2-complex during 100 ns MD Simulations.

The binding affinity of ‘RBD/ACE2-complex’ with curcumin and catechin scored to be − 7.6 kcal/mol and − 9.1 kcal/mol, respectively. Results of the present investigation suggest that amino acid residues of both the components of ‘RBD/ACE2-complex’ that interact with catechin and curcumin are different. While curcumin binds with the ACE-2 receptor in the complex through van der Waals interactions (Phe390, Asn33, Glu37, His34, Asp38, Lys353, Arg396, Pro389), catechin interacts with ACE2 receptor in the complex through van der Waals interactions (Arg559, Gln338, Ser536, Thr92, Leu29, Val93, Lys26, Asn33, Ala386 and His34), conventional hydrogen bonds (Arg393, Glu37, Gln96), carbon–hydrogen bond (Ala387), pi–alkyl bond (Pro389) and pi donor hydrogen bond (Gln96). Curcumin is also engaged with the S Protein segment of the complex through van der Waals interactions (Arg408, Gly416, Ile418, Tyr505, Gln493, Gly496, Tyr495, Arg403), conventional hydrogen bonds (Gln409, Ser494) (Table 4), carbon–hydrogen bond (Glu406); pi–alkyl bond (Lys417), unfavourable donor–donor (Gln409) and pi donor hydrogen bond (Tyr453). The interaction of catechin with the S Protein region of the complex involves van der Waals interaction (Gly416, Gln409, Asp405, Leu455, Tyr453), conventional hydrogen bonds (Glu406, Arg408, Tyr505) and pi–alkyl bond (Lys417) (Table 4). The number of hydrogen bonds formed during interaction of curcumin and catechin with ‘RBD/ACE2-complex’ are 3 and 7, respectively. Such H-bonds are formed between the amino acid residues of the protein complex and OH groups of the polyphenols. Catechin shows high binding affinity towards the receptor due to presence of more number of functional OH groups. The average RMSD of the protein complex (S Protein and ACE2) with catechin and curcumin was calculated as 1.8 Å (Fig. 7), and 2.0 Å (Fig. 8), respectively. The extent of fluctuation caused in the protein complex due to binding of catechin and curcumin was 3.3 Å and 4.2 Å, respectively. Further, these two polyphenols are also able to cause fluctuation in both alpha-helical and beta-strand regions of the complex. While curcumin-mediated fluctuation is more localized to S Protein part of the complex than ACE2, catechin-mediated fluctuation is observed in both the components of the complex (Fig. 9). The maximum and the minimum MM-GBSA binding energies for ‘RBD/ACE2-complex’ with curcumin were found to be − 69 kcal/mol (at 10 ns) and − 53 kcal/mol (at 90 ns), respectively. Similarly, during interaction of ‘RBD/ACE2-complex’ with catechin, the maximum and the minimum MM-GBSA binding energies were found to be − 57 kcal/mol (at 20 ns) and − 28 kcal/mol (at 80 ns), respectively (Supplementary Fig. S15). Total MM-GBSA free energy calculation indicates a favourable binding energy for the interaction of curcumin with ‘S Protein and ACE2 complex’ (− 60.84 kcal/mol) as compared to that of catechin (− 45.04 kcal/mol). Upon docking of both polyphenols on the interface of ‘RBD/ACE2-complex’ concomitant with 100 ns MD simulation, it was portrayed that stable and favourable interactions are formed by the polyphenols with both RBD and ACE2 proteins. Further, a higher binding affinity of both polyphenols was observed towards RBD than ACE2.

**Figure 10:**
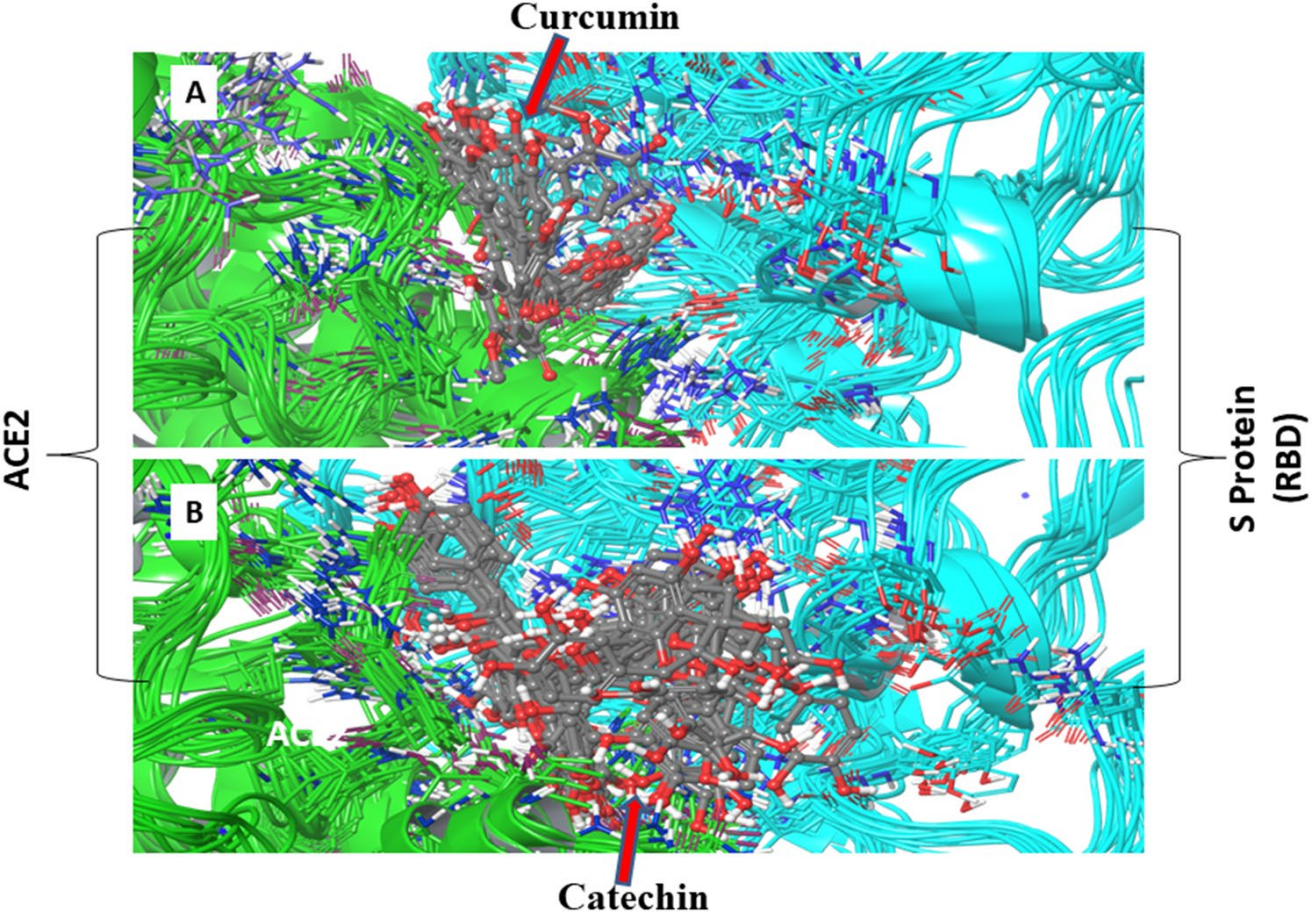
Superimposition of mutliple snapshots taken during interaction of (**A**) curcumin and (**B**) catechin with RBD/ACE2-complex, derived from the 100 ns of MD simulations at 10 ns interval.

Results of the present study suggest that amino acid residues of both the components of ‘RBD/ACE2-complex’ that interact with polyphenols used in the present study considerably differ depending on the nature of polyphenols as well as the components of the complex. In addition, interaction time of amino acid residues of both the components of the complex varies depending upon the types of polyphenol. For example, the residues like Glu37 and Arg393 of ACE2 receptor of the complex interact with catechin through conventional hydrogen bond for 97% and 94% of the simulation time, respectively, while Ala387 interacts for 87% of simulation time through carbon-hydrogen bond. Similarly, amino acid residues like Asp38 of ACE2 and Gly496 of S Protein of the complex are engaged with curcumin for 94% and 83% of the total simulation time i.e. 100 ns, respectively, through van der Waals interaction (Supplementary Fig. S13). It is worth mentioning over here that all interactions are above 80% of the total simulation trajectory. The information derived above from simulation study is also supported by well-aligned superimposition of curcumin and catechin on RBD of ‘S Protein and ACE2-complex’ during 0–100 ns simulation^38^ (Fig. 10). The docking and simulation outcomes depict high binding energies which reflect that these two polyphenols bind strongly in the interface of ‘RBD/ACE2-complex’.

It is observed that the binding between S Protein (RBD)—ACE2 complex (Supplementary Figs. S17, S18) has different bonding patterns (H-bonds, Hydrophobic and salt bridge interactions) where count and the intensity of interacting amino acid residues for Apo Chain A and B with respect to each other is relatively high. In contrast, the presence/binding of catechin or curcumin in the interface of ‘RBD/ACE2-complex’ minimises such interactions.

Results obtained from multiple evidences depicted that both the polyphenols bind preferably to sites of S Protein (RBD site) which are crucial in host cell binding^33^. Similarly, it was also seen that these molecules attach to those sites of ACE2 which were involved in serving a medium of viral entry^39^. Thus, results of the present computational studies suggest possible prevention of the viral infection by the use of catechin and curcumin, two widely used natural polyphenols. This dual inhibitory machinery of blocking the binding of host cell receptors to virus and inhibiting cellular entry of viral protein could be an effective therapeutic target, as evident from an array of computational studies. However, this needs to be experimentally validated prior to translational intervention.

In addition, elimination and neutralization of viral infection by catechin and curcumin cannot be ignored because both the polyphenols are well acclaimed immuno-stimulant and inducer of autophagy, another important mechanism of viral clearance^14,40^.

Therefore, availability of catechin and curcumin in the host system may facilitate all different mechanisms simultaneously and, thereby promote elimination and/or neutralisation of viral infection.

## Conclusion

The pandemic novel corona virus has created a stark landscape in the social, health and economic sphere. The lethality of the virus has taken many lives. There is urgency to curb the widespread outbreak of SARS-CoV2. In this context, findings of this computational study indicate that catechin and curcumin can be considered for prospective antiviral drugs against SARS-CoV2. Nevertheless, this requires further experimental validation to substantiate the findings.

## Supplementary Information

Supplementary Information

## Data Availability

The data relevant to illustrative structures and interactive versions of Figs. 2, 3, 4, 5, 6, 10, S2, S3, S4, S8, S9 and S16 have been submitted to figshare (https://doi.org/10.6084/m9.figshare.13379453).
