# Supplementary material for: Catechin and curcumin interact with S protein of SARS-CoV2 and ACE2 of human cell membrane: insights from computational studies
Source: Sci Rep. 2021 Jan 21;11:2043. doi: 10.1038/s41598-021-81462-7 (PMC7820253; doi:10.1038/s41598-021-81462-7)
Supplement: Supplementary file 1 — Supplementary Information [file 41598_2021_81462_MOESM1_ESM.docx]

**Catechin and Curcumin interact with S protein of SARS-CoV2 and ACE2 of human cell membrane: Insights from Computational studies**

**Atala B. Jena^a^, Namrata Kanungo^b^, Vinayak Nayak^b^, G.B.N. Chainy^b^, Jagneshwar Dandapat^a,b^***

**^a^Centre Of Excellence In Integrated Omics and Computational Biology, Utkal University, Bhubaneswar 751004, Odisha.**

**^b^Post Graduate Department of Biotechnology, Utkal University, Bhubaneswar 751004, Odisha.**

***Corresponding Author: jdandapat.nou@gmail.com, jd.biotech@utkaluniversity.ac.in**

**(Prof. J. Dandapat)**

**
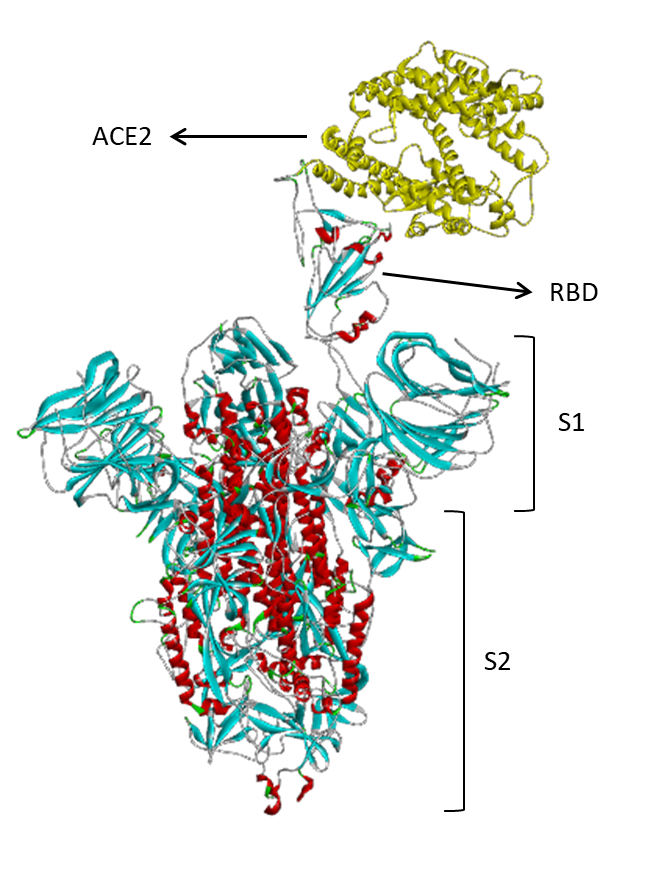
**

**Figure S1. Structure-based representation of S Protein and ACE2 complex, depicting their interaction through RBD (Receptor Binding Domain) of S-protein. This figure has been developed using Discovery Studio Visualizer (http://accelrys.com/products/collaborative-science/biovia-discovery-studio/ visualization-download.php).**

**
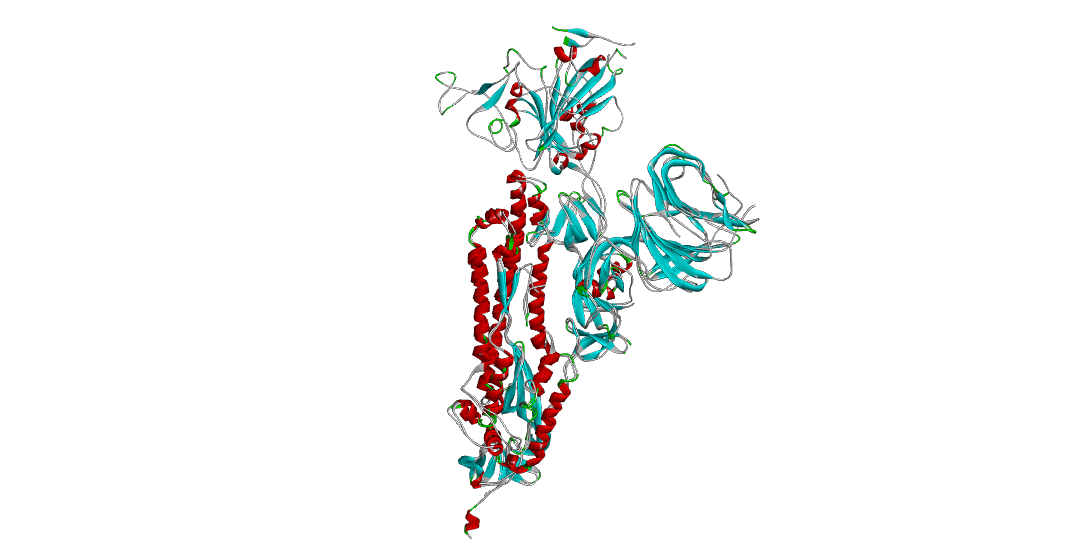
**

**Figure S2. Structure-Structure alignment by superimposition of S Protein of SARS-CoV2 on SARS-CoV. This figure has been developed using TM-align (https://zhanglab.ccmb. med. umich.edu/TM-align/).**

**
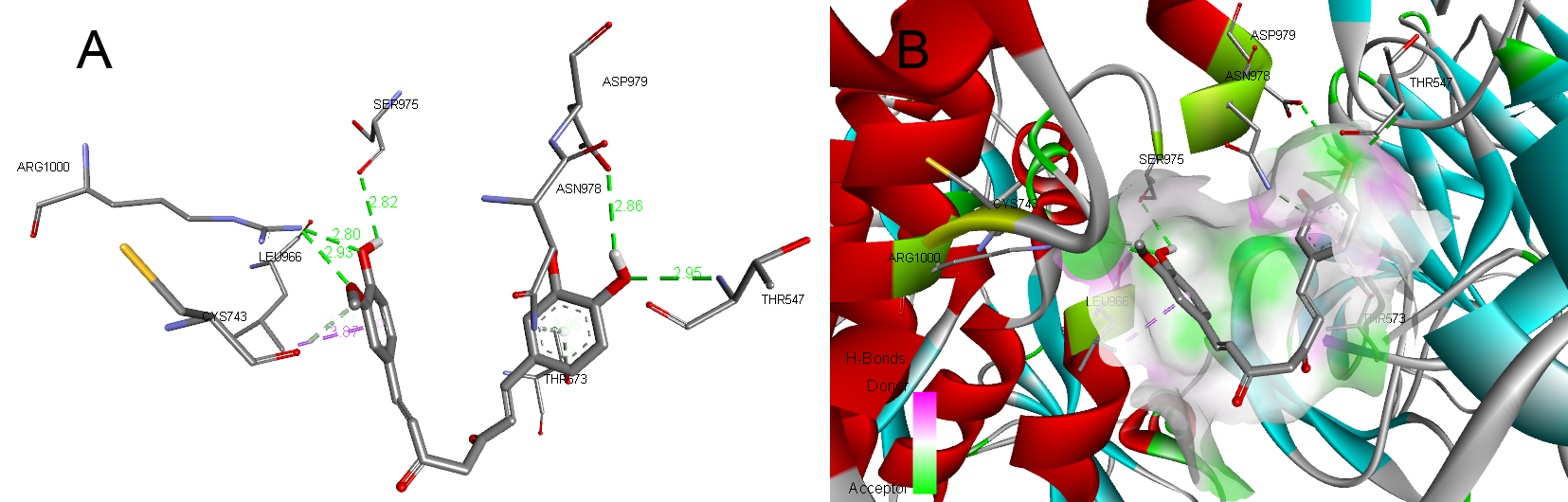
**

**Figure S3. Docked pose of curcumin in the binding pocket of S Protein. (A) Participating amino acids in the interaction of curcumin and S Protein, (B) Several bonds between curcumin and S Protein in Binding pocket. This figure was produced using Discovery Studio Visualizer (**[**http://accelrys.com/products/collaborative-science/biovia-discovery-studio/**](http://accelrys.com/products/collaborative-science/biovia-discovery-studio/) **visualization-download.php)**

**
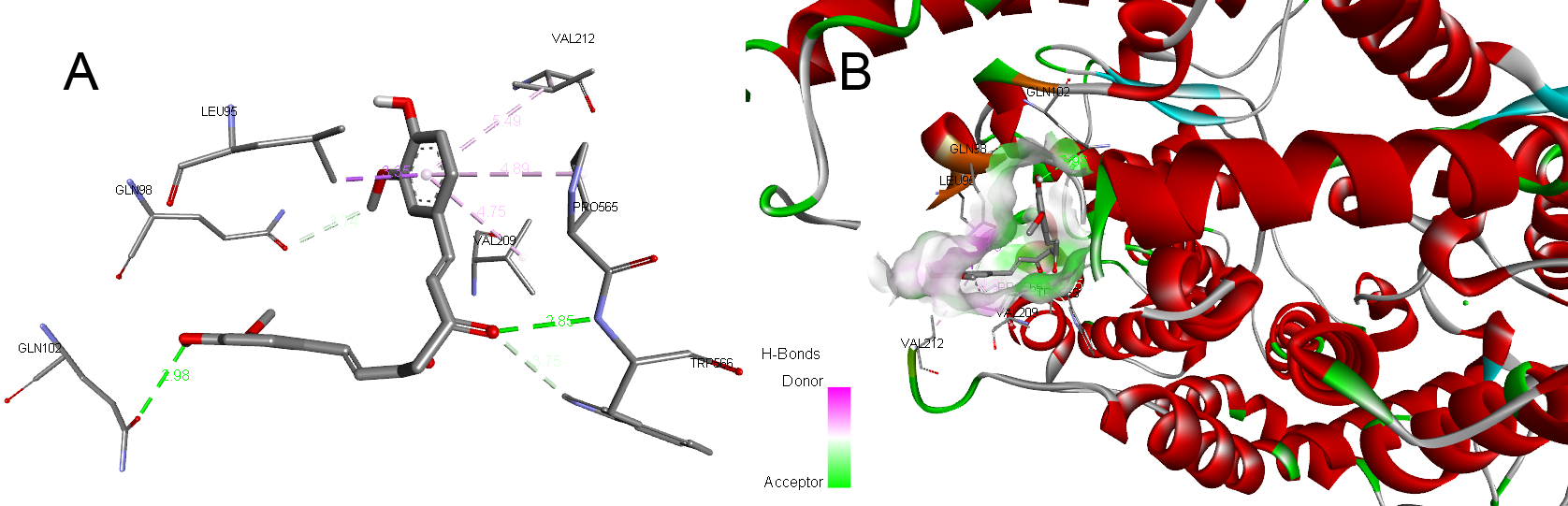
**

**Figure S4. Docked pose of curcumin in the binding pocket of ACE2 Receptor. (A) Participating amino acids in the interaction of curcumin and ACE2 Receptor, (B) Several bonds between curcumin and ACE2 Receptor. This figure has been developed using Discovery Studio Visualizer (**[**http://accelrys.com/products/collaborative-science/biovia-discovery-studio/**](http://accelrys.com/products/collaborative-science/biovia-discovery-studio/) **visualization-download.php).**

**
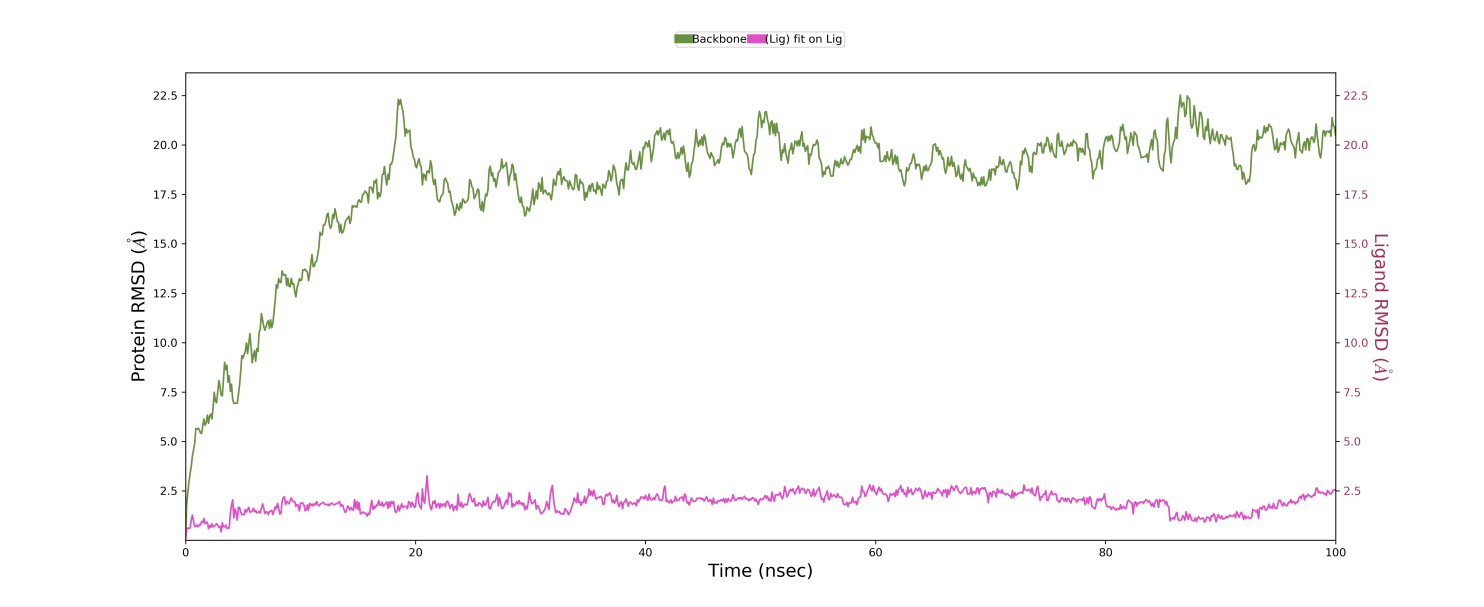
**

**Figure S5. Root Mean Square Deviation (RMSD) plot for interactive complex of curcumin and S Protein during 0 – 100 ns of molecular dynamic simulation.**

**
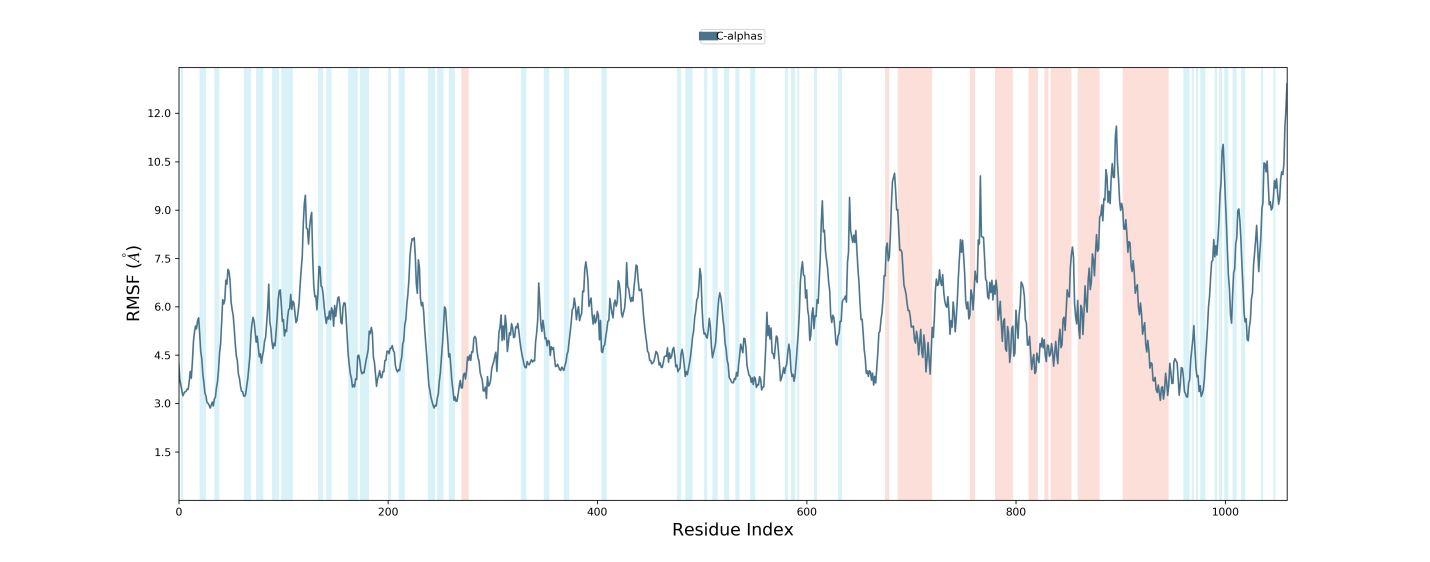
**

**Figure S6. RMSF plot depicting curcumin induced fluctuations in the entire amino acid sequence of S Protein during 100ns MD Simulations.**

**
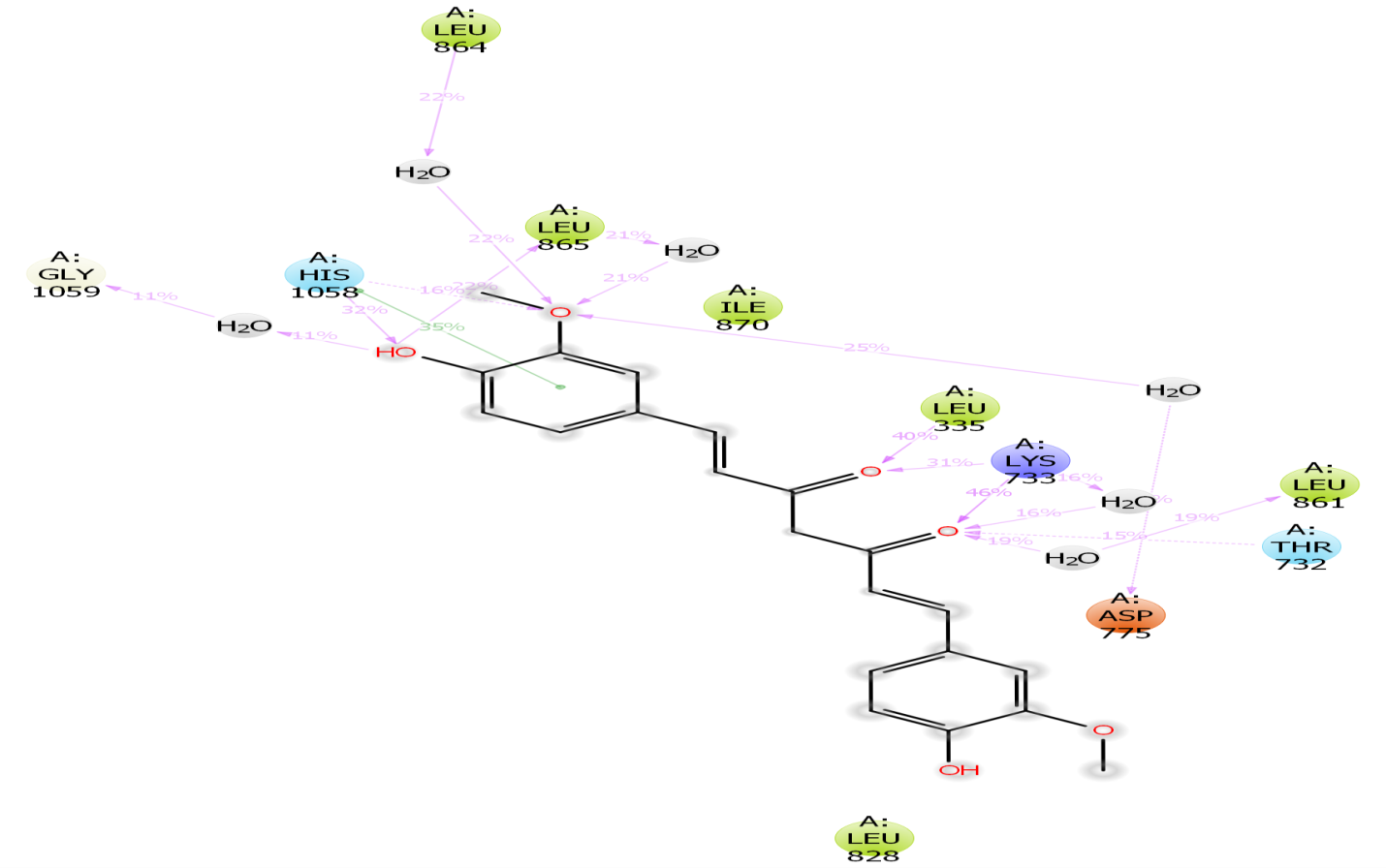
**

**Figure S7. Illustration of bonds between amino acid residues of S Protein and curcumin interaction during 100 ns simulation trajectory.**

**
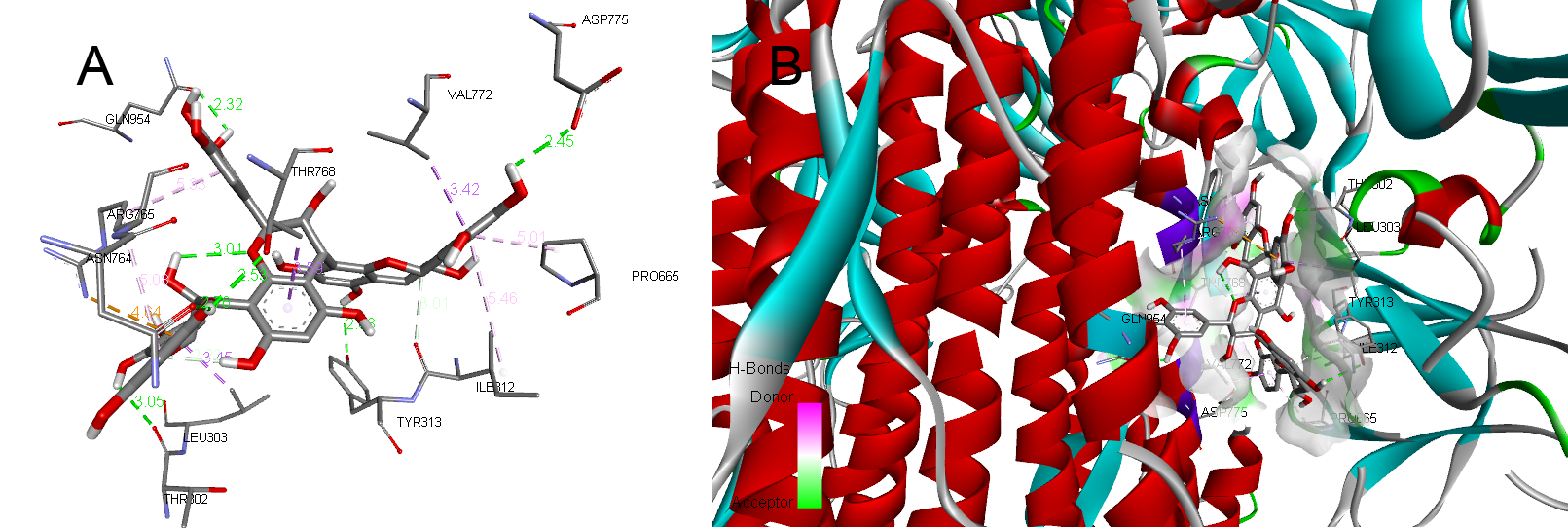
**

**Figure S8. Docked pose of catechin in the binding pocket of S Protein. (A) Participating amino acids in the interaction of catechin and S Protein, (B) Several bonds between catechin and S Protein in Binding pocket. This figure was produced using Discovery Studio Visualizer (**[**http://accelrys.com/products/collaborativescience/biovia-discovery-studio/**](http://accelrys.com/products/collaborativescience/biovia-discovery-studio/) **visualization-download.php).**

**
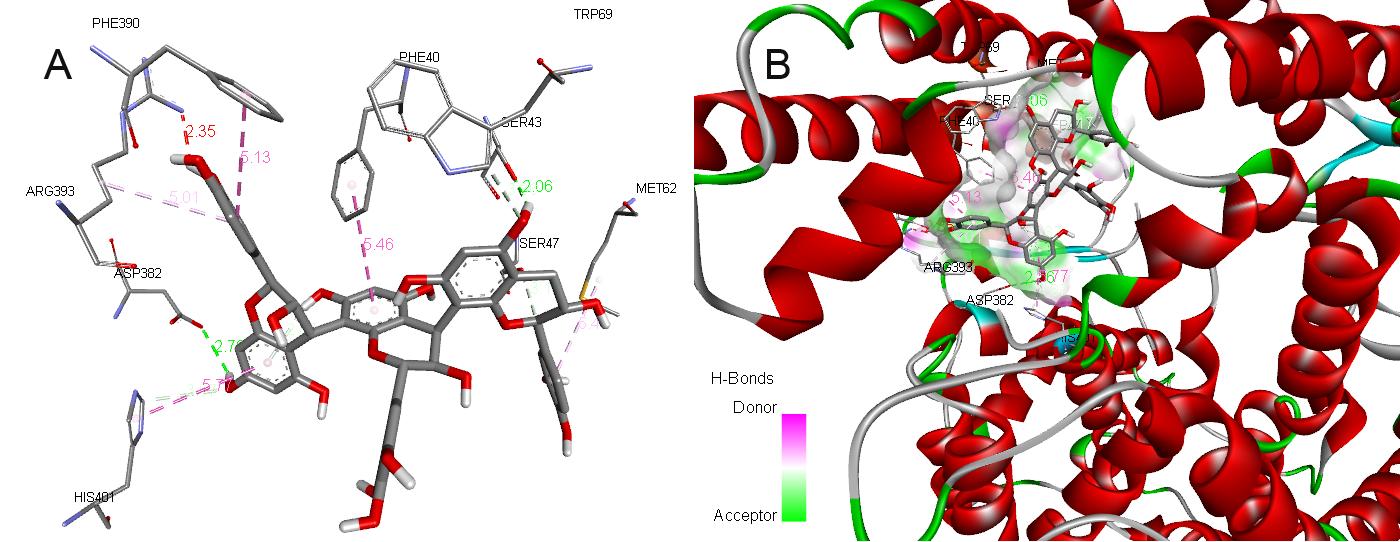
**

**Figure S9. Docked pose of catechin in the binding pocket of ACE2 Receptor. (A) Participating amino acids in the interaction of curcumin and ACE2 Receptor, (B) Several bonds between catechin and ACE2 Receptor. This figure has been developed using Discovery Studio Visualizer (**[**http://accelrys.com/products/collaborativescience/biovia-discovery-studio/**](http://accelrys.com/products/collaborativescience/biovia-discovery-studio/) **visualization-download.php).**

**
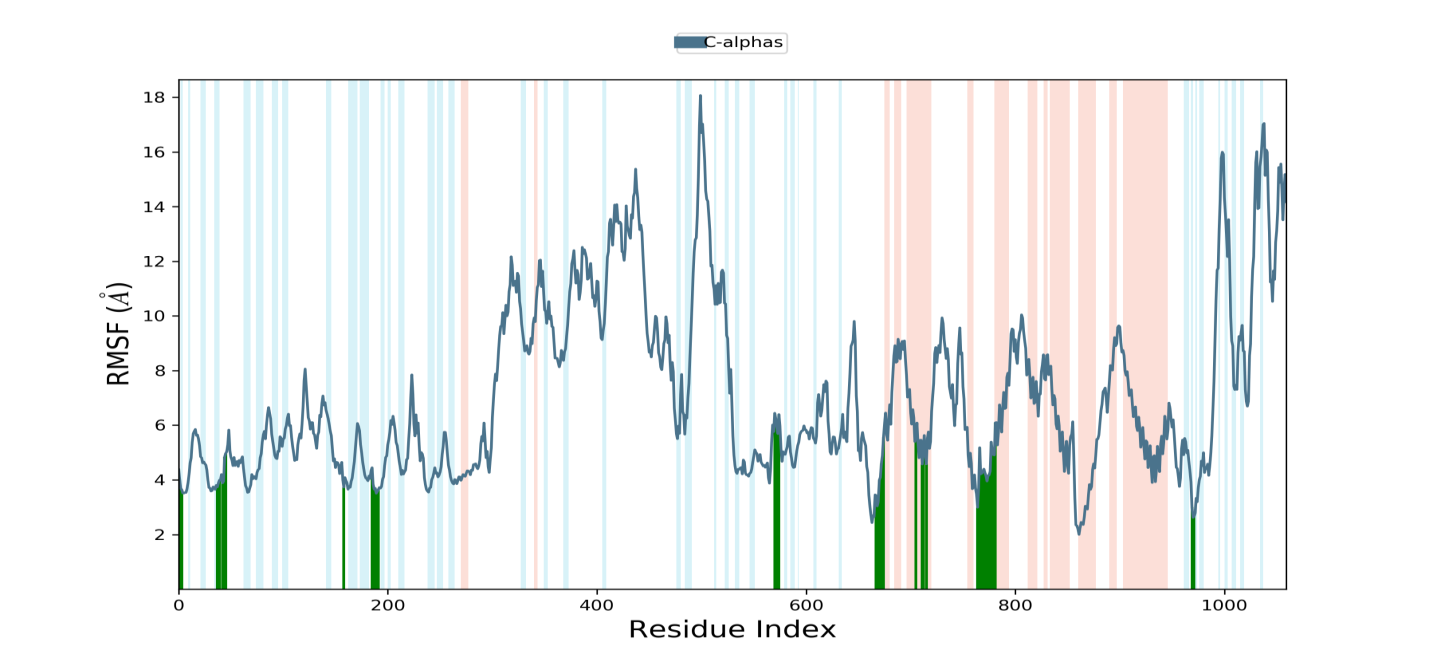
**

**Figure S10. RMSF plot depicting catechin induced fluctuations in the entire amino acid sequence ofSprotein during 100ns MD Simulations.**

**
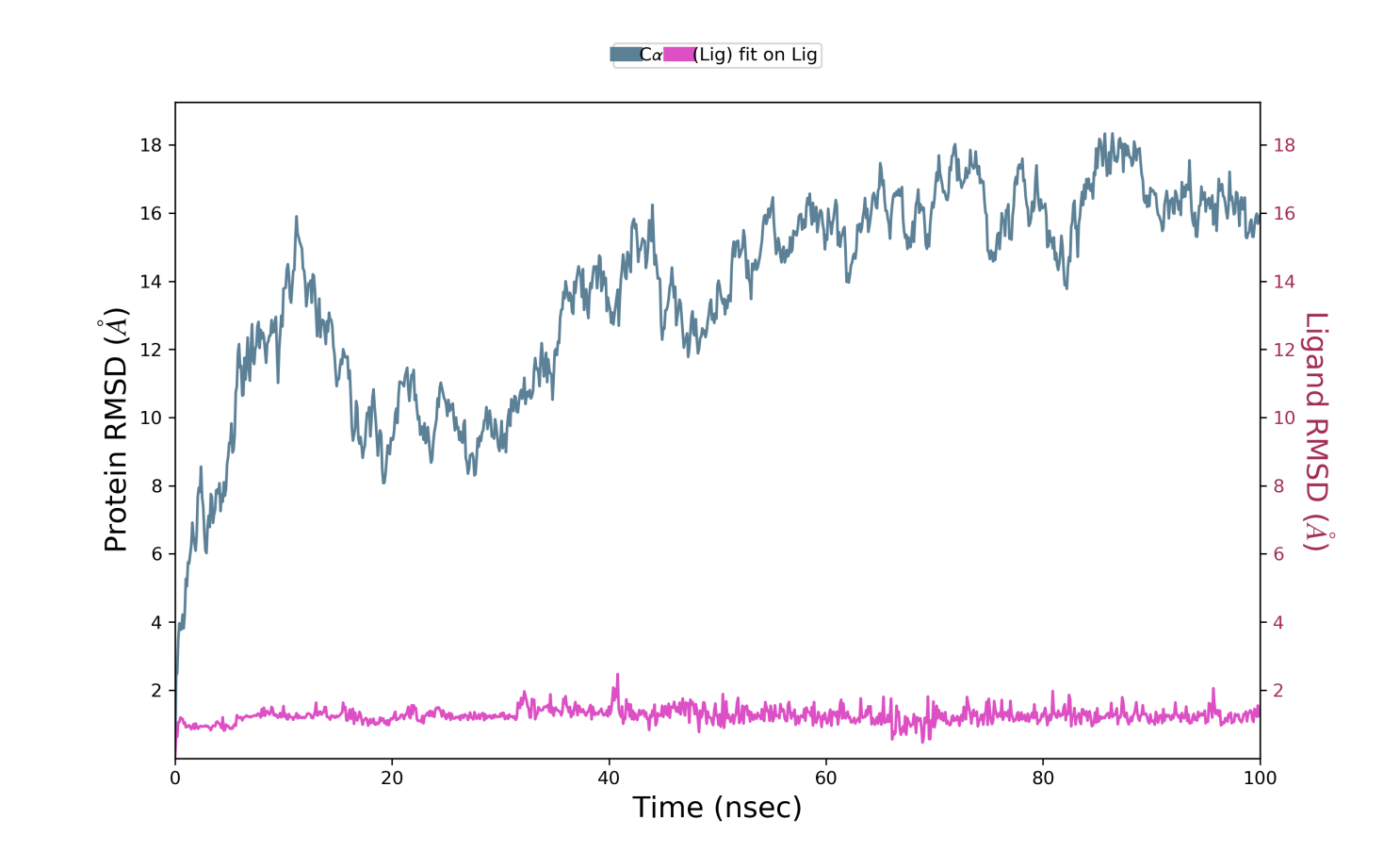
**

**Figure S11. Root Mean Square Deviation (RMSD) plot for interactive complex of catechin and S Protein during 0 – 100 ns of molecular dynamic simulation.**

**
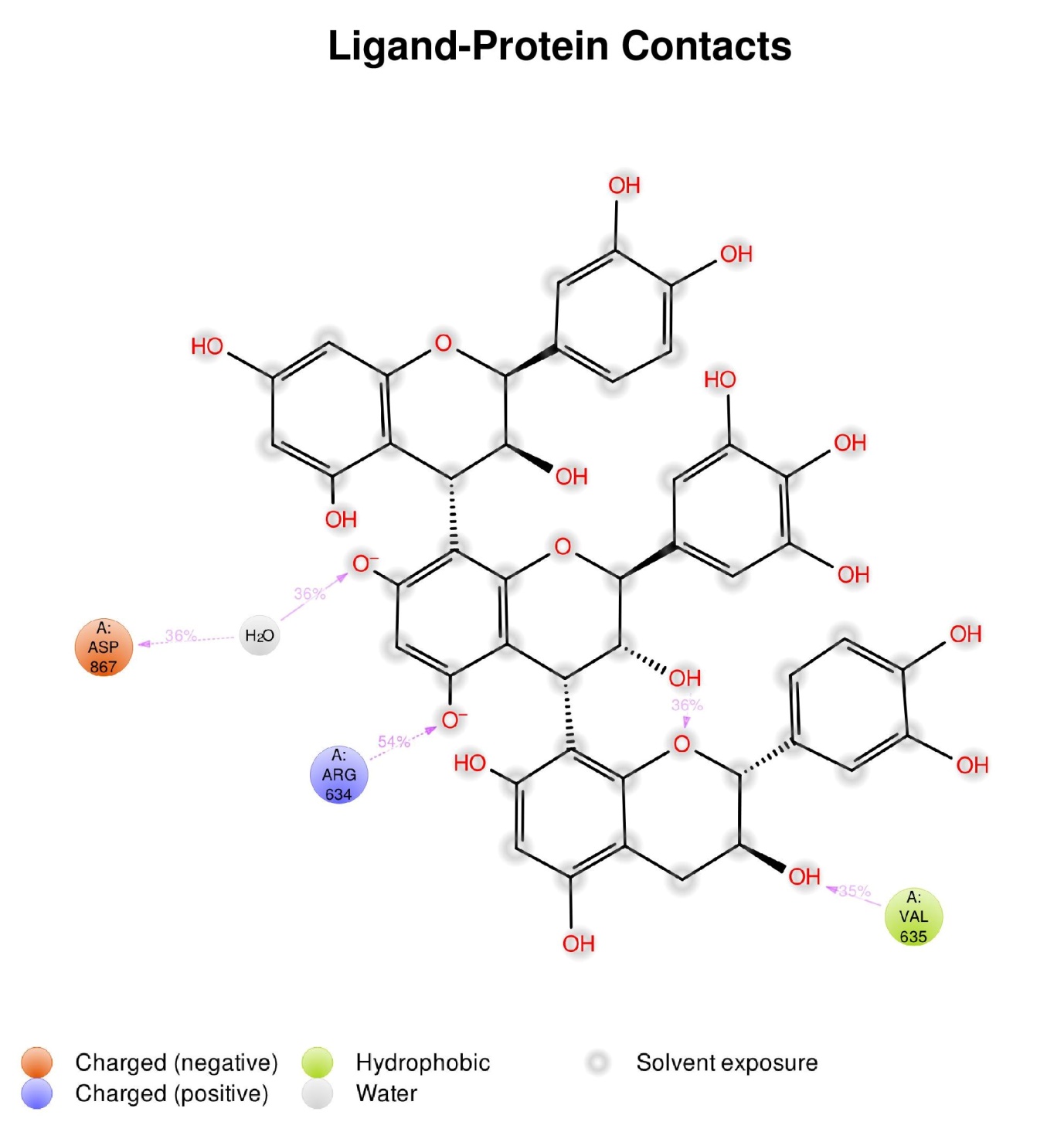
**

**Figure S12. Illustration of bonds between amino acid residues of S Protein and catechin with more than 30% interaction timeduring the simulation period.**

**
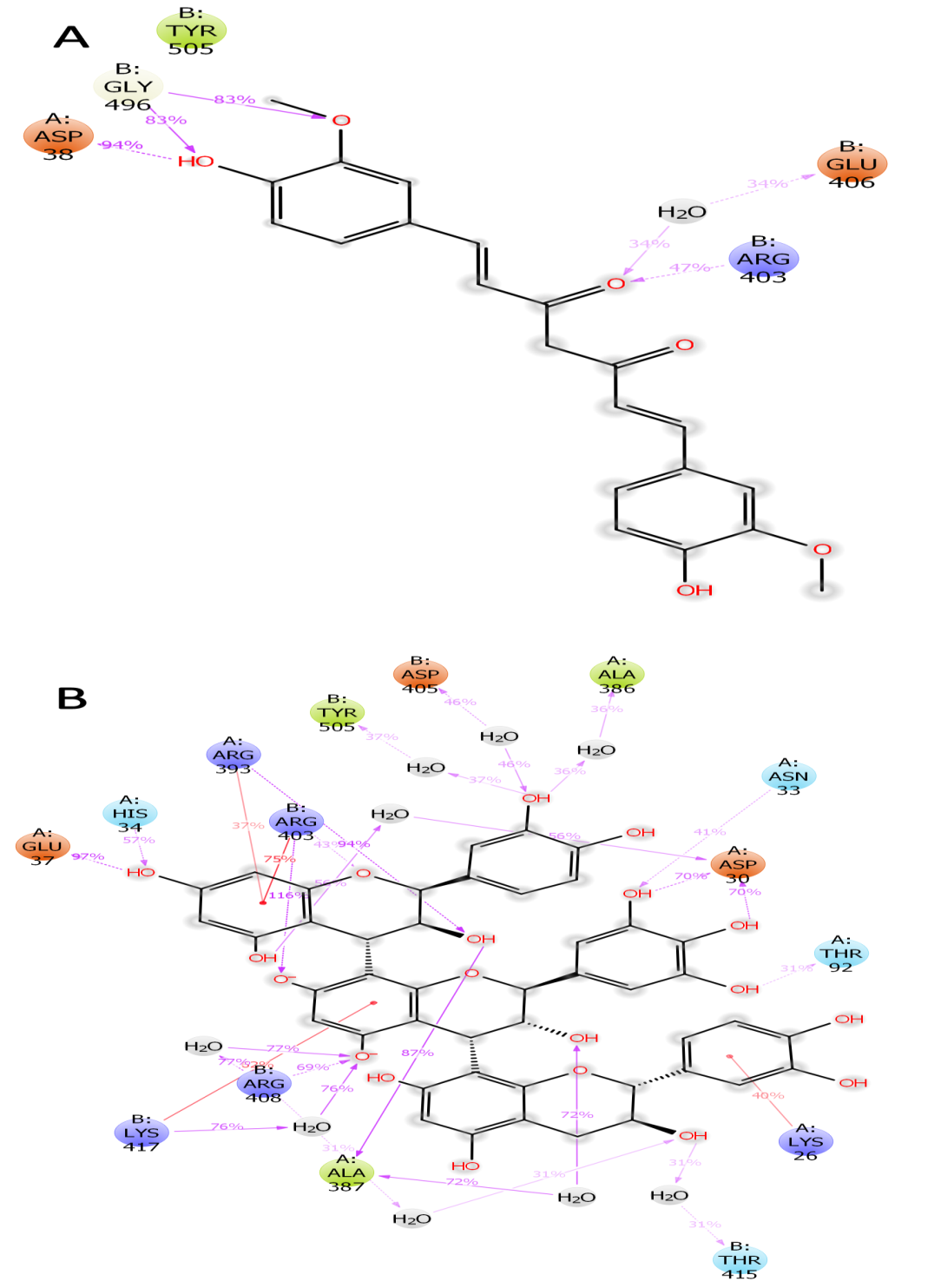
**

**Figure S13. (A) Illustration of bonds between amino acid residues of RBD/ACE2 - complex and curcumin engaged for more than 30% of interaction period during 100ns simulation trajectory. (B) Illustration of bonds between amino acid residues of RBD/ACE2 - complex and catechin with more than 30% interaction time during 100 ns simulation trajectory.**

**
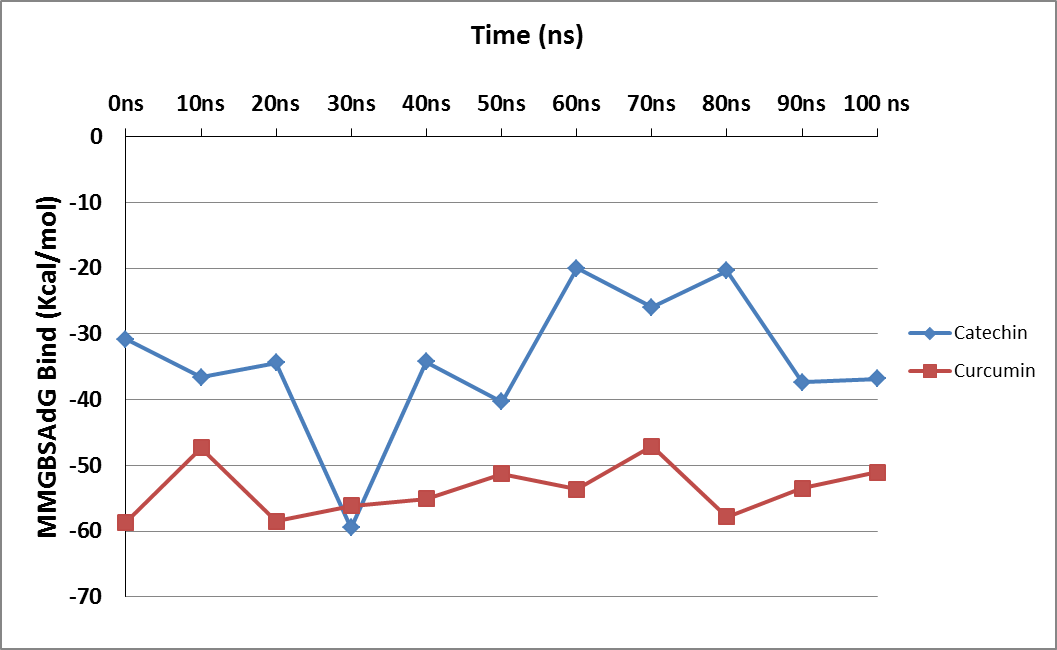
**

**Figure S14. MMGBSA binding energy of curcumin-S Protein and catechin-S Protein complexes.**

**
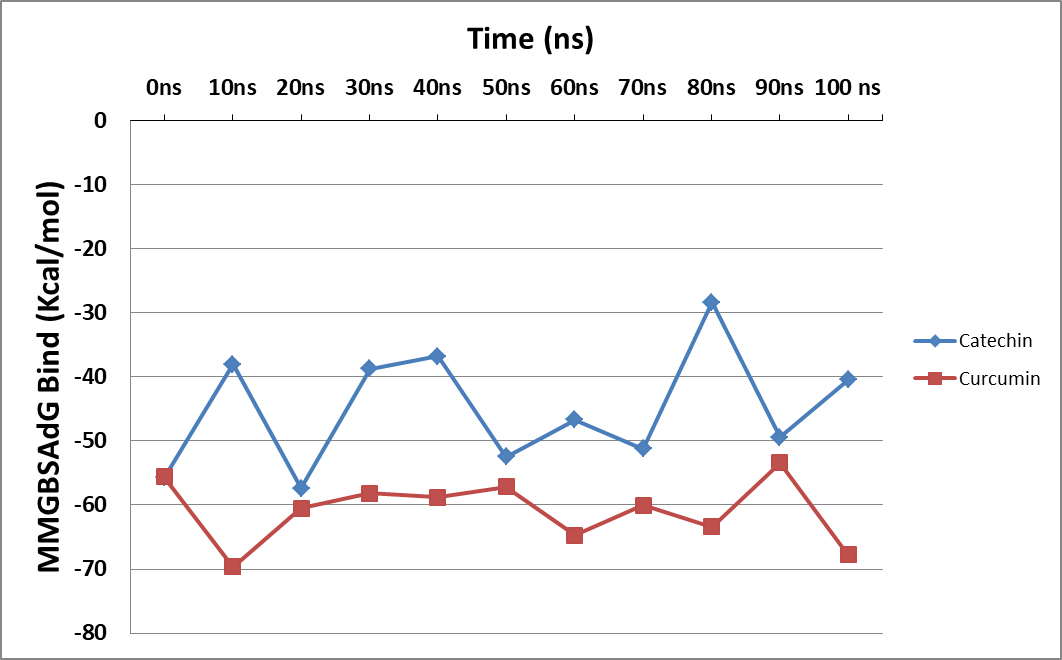
**

**Figure S15. MMGBSA binding energy of curcumin with RBD/ACE2 - complex and catechin-with RBD/ACE2 - complex.**

**
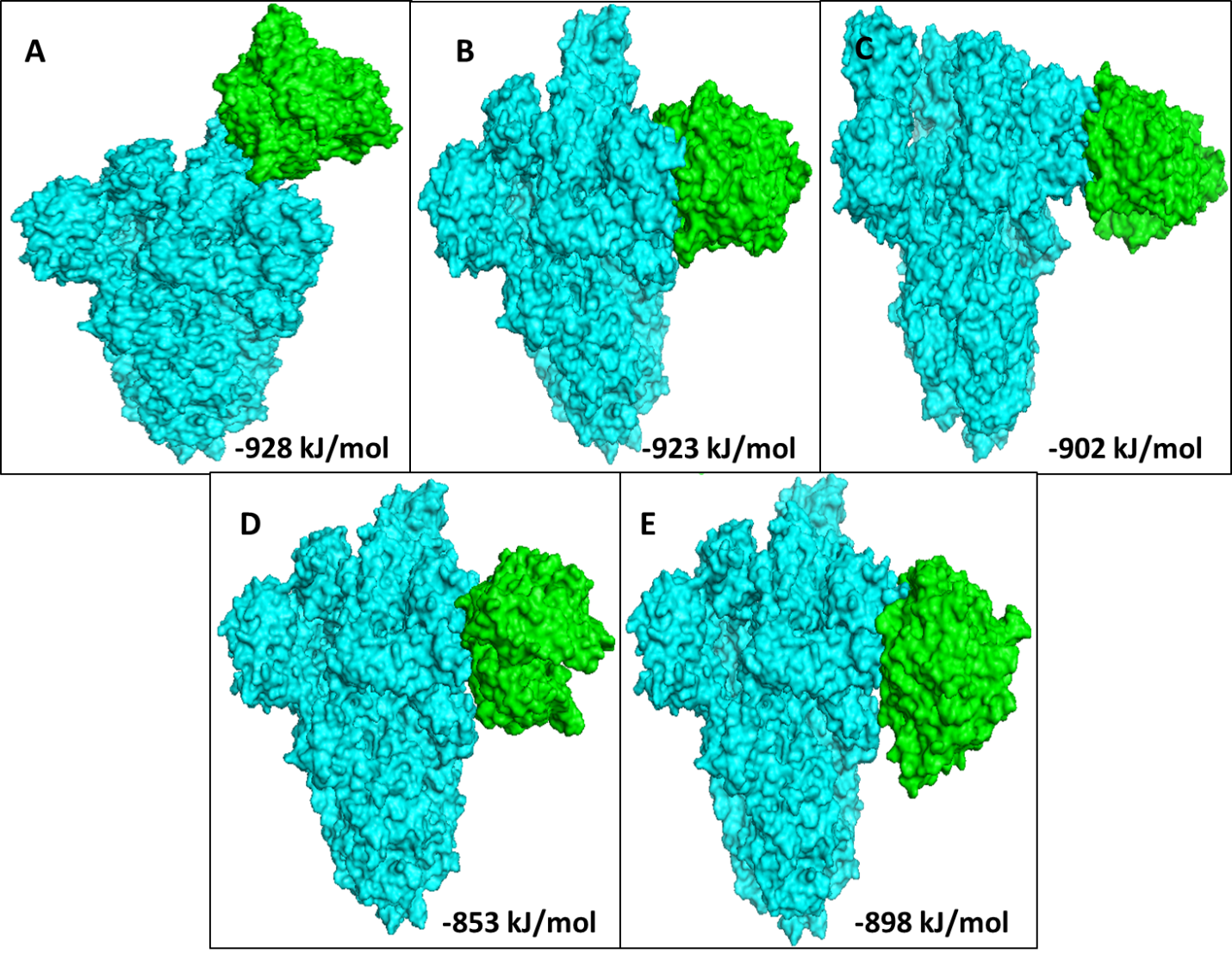
**

**Figure S16. The top 5 docked models displaying interaction of S Protein with ACE2 receptor.**

**
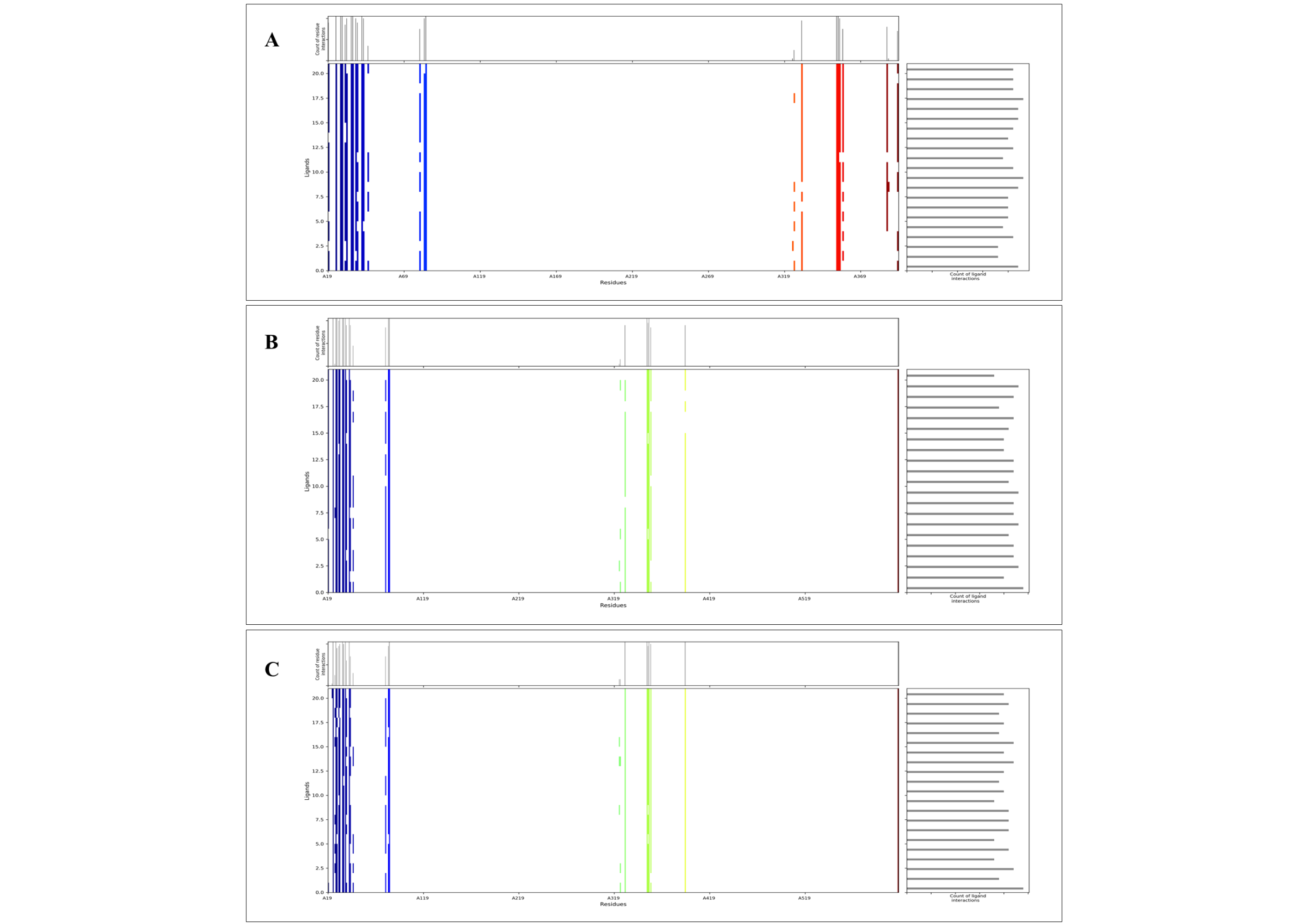
**

**Figure S17. Non Bonding interaction qualitative analysis for protein-protein binding for bound and unbound chain B against chain A. (A) Absence of any phytocompound. (B) Presence of curcumin. (C) Presence of catechin.**

**
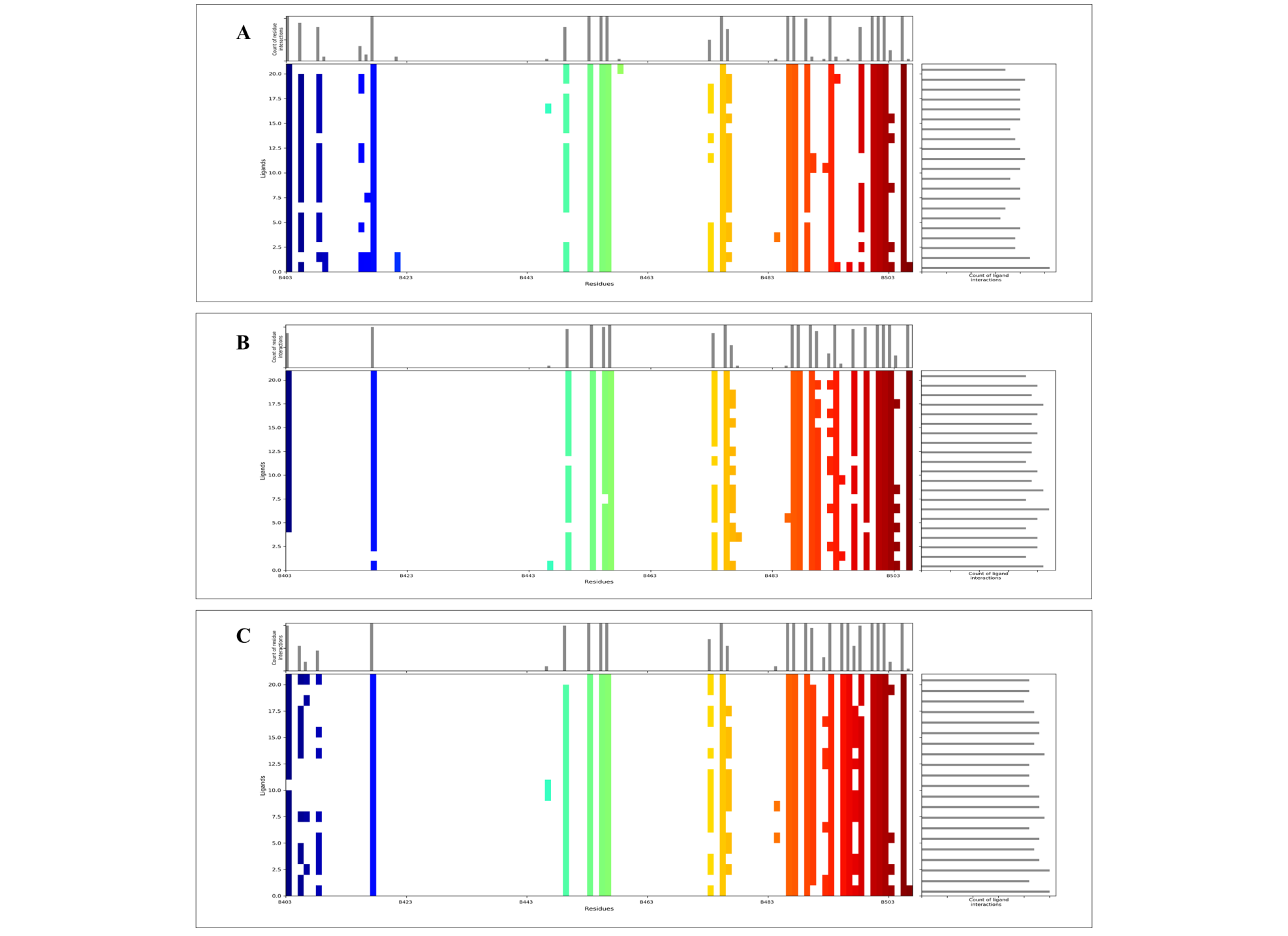
**

**Figure S18. Non Bonding interaction qualitative analysis for protein-protein binding for bound and unbound chain A against chain B. (A) Absence of any phytocompound. (B) Presence of curcumin. (C) Presence of catechin.**

**Table S1. Interacting amino acid residues of S Protein with curcumin during 100 ns simulation trajectory.**

| **Aminoacid residue of S Protein** | **Resident time in ns** |
| --- | --- |
| **THR333** | **14** |
| **ASN334** | **24** |
| **LEU335** | **65** |
| **CYS336** | **1** |
| **PRO337** | **8** |
| **PHE338** | **1** |
| **GLY339** | **3** |
| **GLU340** | **14** |
| **ASP364** | **1** |
| **LYS529** | **1** |
| **VAL729** | **26** |
| **SER730** | **48** |
| **MET731** | **43** |
| **THR732** | **44** |
| **LYS733** | **91** |
| **ASP775** | **41** |
| **THR778** | **42** |
| **PHE782** | **2** |
| **PHE823** | **4** |
| **VAL826** | **5** |
| **THR827** | **18** |
| **LEU828** | **70** |
| **GLN853** | **27** |
| **LYS854** | **25** |
| **VAL860** | **21** |
| **LEU861** | **31** |
| **PRO862** | **1** |
| **PRO863** | **30** |
| **LEU864** | **31** |
| **LEU865** | **36** |
| **ASP867** | **24** |
| **ILE870** | **27** |
| **GLN949** | **1** |
| **VAL952** | **29** |
| **ASN953** | **1** |
| **ASN955** | **8** |
| **ALA956** | **16** |
| **LEU959** | **15** |
| **ASN960** | **17** |
| **SER1055** | **22** |
| **ALA1056** | **37** |
| **PRO1057** | **3** |
| **HIS1058** | **78** |
| **GLY1059** | **26** |
